# Supplementary material for: Application of high-throughput single-nucleus DNA sequencing in pancreatic cancer
Source: Nat Commun. 2023 Feb 10;14:749. doi: 10.1038/s41467-023-36344-z (PMC9918733; doi:10.1038/s41467-023-36344-z)
Supplement: Supplementary file 1 — Supplementary Information [file 41467_2023_36344_MOESM1_ESM.pdf]

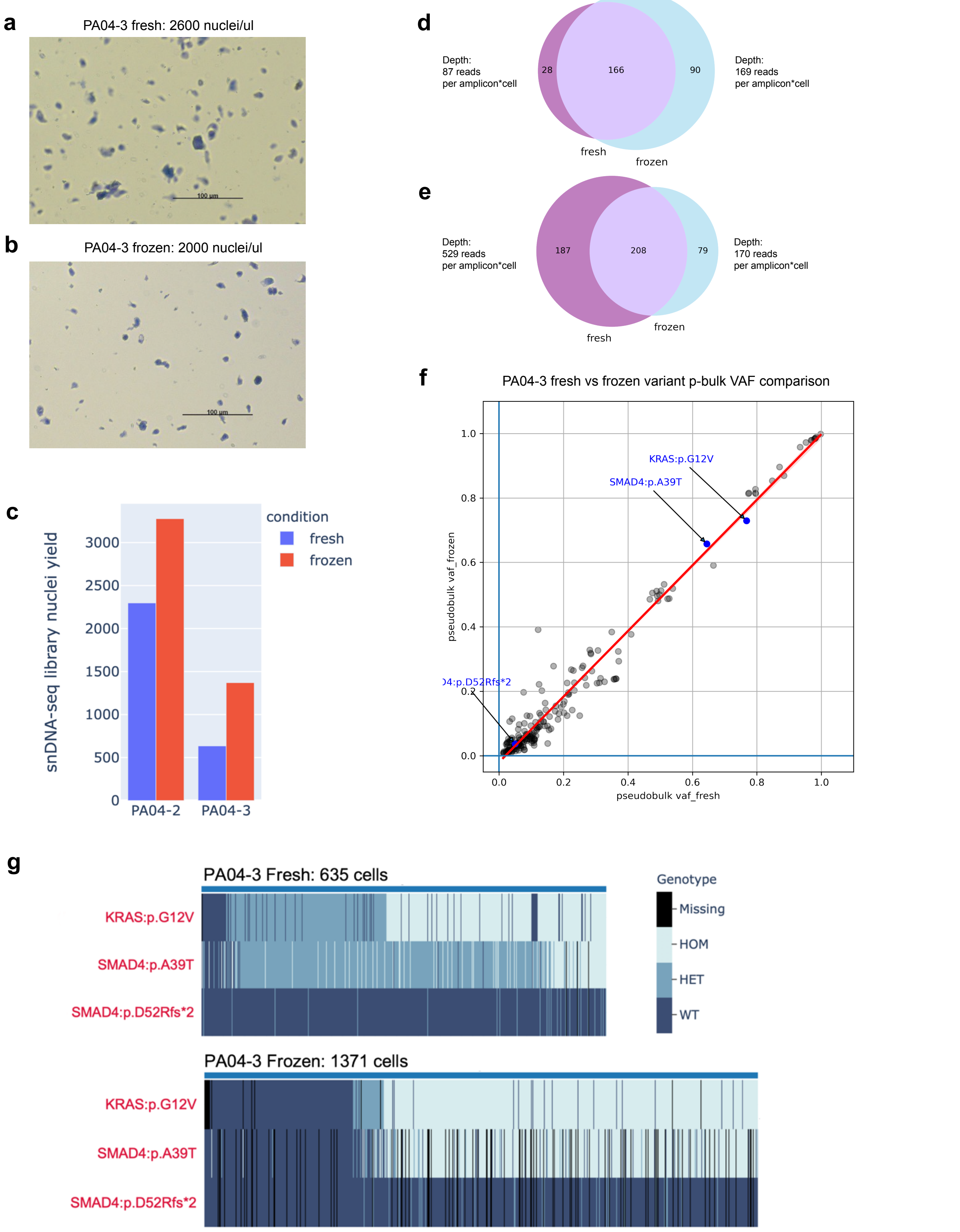

Supplementary figure 1: Nuclei cryopreservation results

a-b. Sample PA04-3 nuclei suspension before **(a)** and after cryopreservation **(b)**. Nuclei were stained with Trypan blue and visualized under brightfield microscope. At least 3 representative pictures were taken per sample and yielded similar results.
c. snDNA-seq library nuclei yield comparison of fresh vs frozen nuclei of the same samples.
d-e. Venn diagram comparing the sets of high-quality variants (**Methods**) identified in snDNA-seq libraries generated by fresh vs frozen (3 weeks) nuclei of sample PA04-2 **(d)**; fresh vs frozen (14 weeks) nuclei of sample PA04-3 **(e)**. aThe mean read depths of each library are labeled.
f. Pseudobulk VAF comparison of all 212 shared variants between libraries generated with fresh vs frozen (14 weeks) nuclei of sample PA04-3. Key drivers preidentified by bulk WES in this case are highlighted; regression line with 90% confidence interval is drawn.
g. Single-cell genotype heatmap of snDNA-seq libraries generated by unsorted vs sorted nuclei of sample PA04-3. The nuclei are sorted based on KRAS variant's variant allele frequency (VAF) in ascending order from left to right.
Source data are provided as a Source Data file.

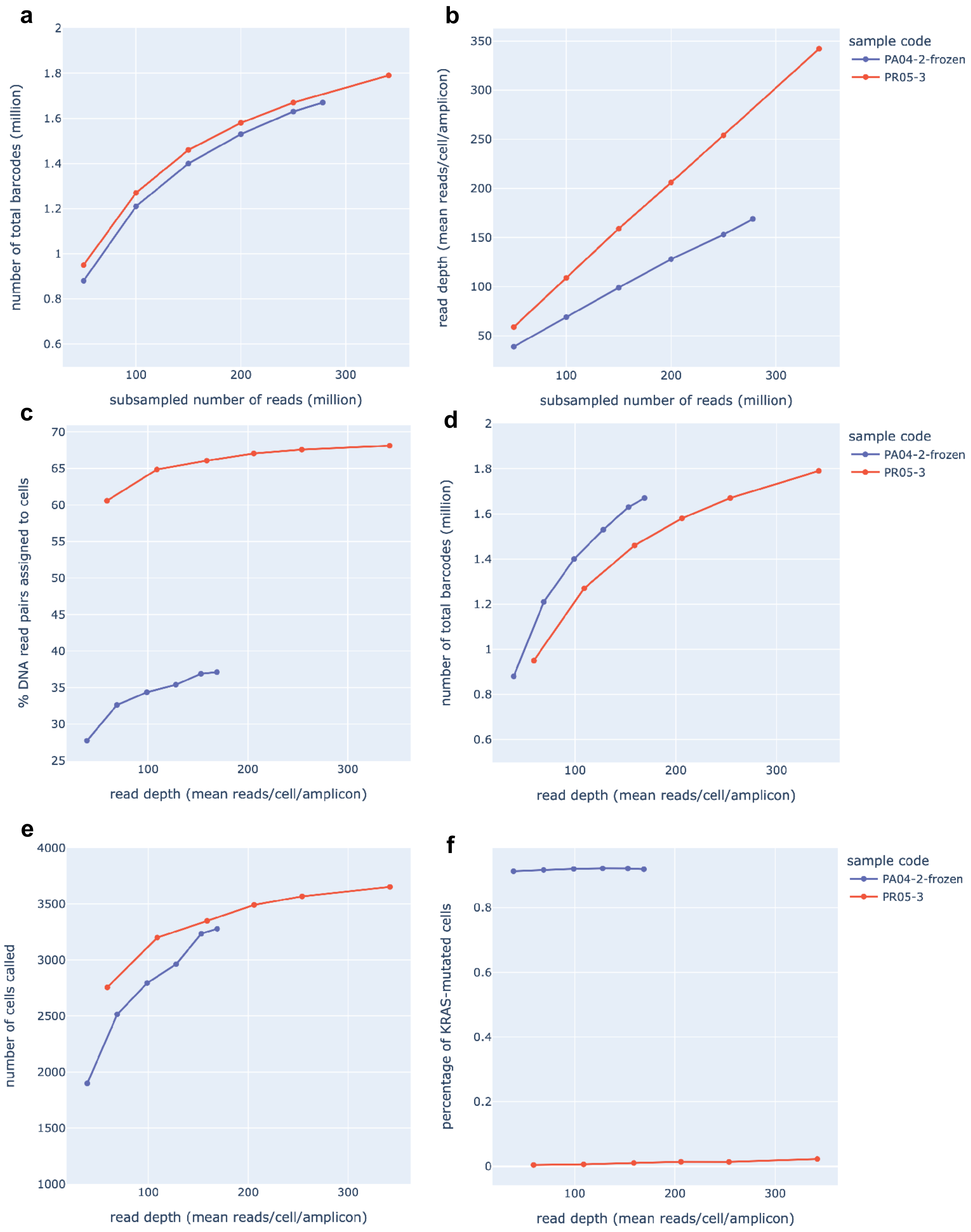

Supplementary Figure 2: subsampling experiment results

Line plots showing correlation between total/mean read depth and several import technical parameters.
a. total number of reads vs. total number of cell barcodes detected.
b. total number of reads vs. mean read depth (per cell per amplicon).
c. mean read depth vs. percentage of DNA read pairs assigned to cells.
d. mean read depth vs. number of total barcodes.
e. mean read depth vs. number of cells called.
f. mean read depth vs percentage of KRAS mutated single nuclei.

Source data are provided in **Supplementary Table 4.**

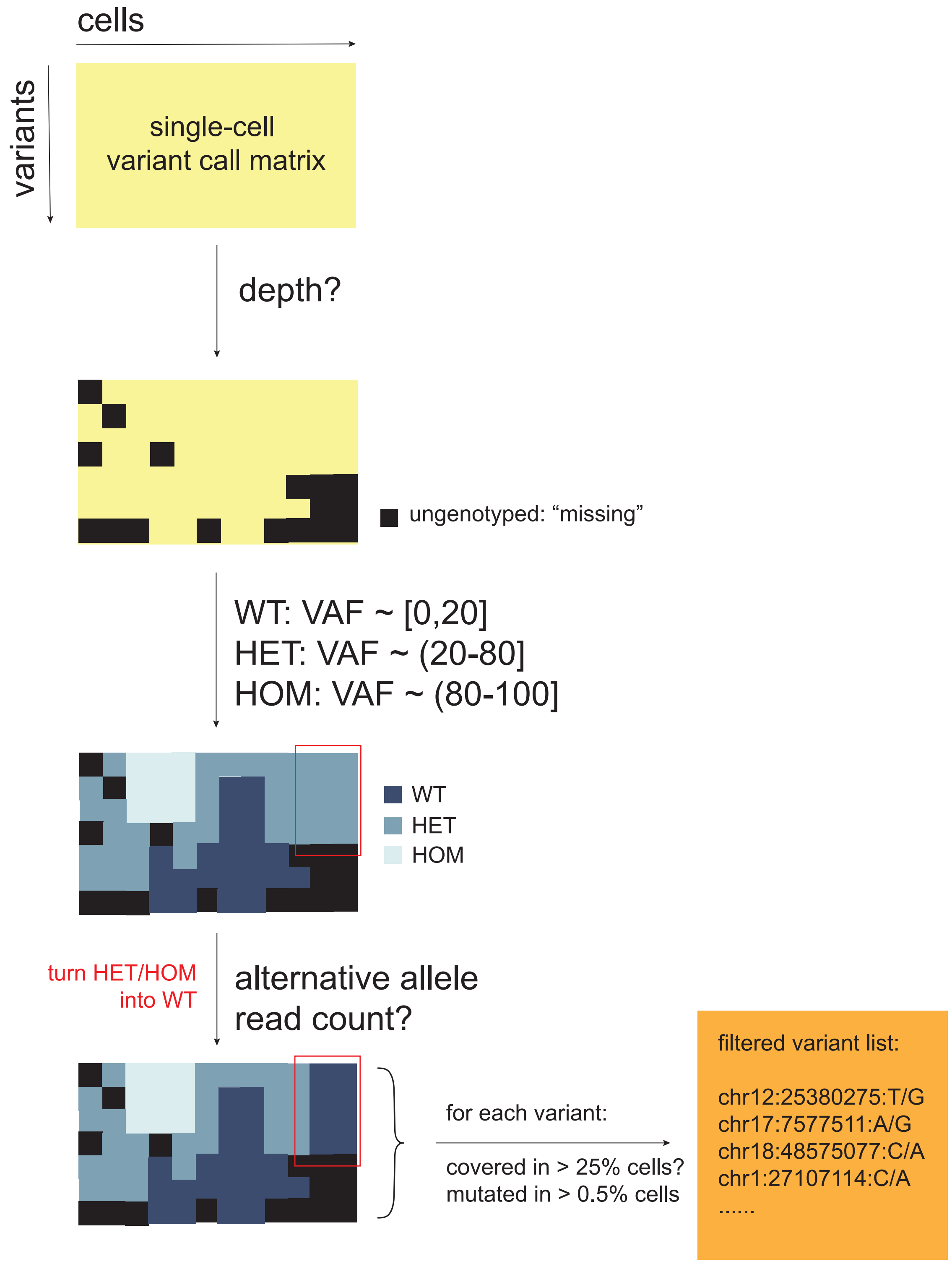

Supplementary figure 3: single-cell variant matrix genotyping and filtering procedure

To enable comparison of variant call sets across technical replicates, the genotyping and filtering scheme outlined in the figure and detailed in the method section was used. Briefly, each variant in each single cell was first genotyped with read depth and variant allele frequency (VAF) thresholds, and then hard filtered based on coverage and mutational prevalence to result in a final call set for each sample.

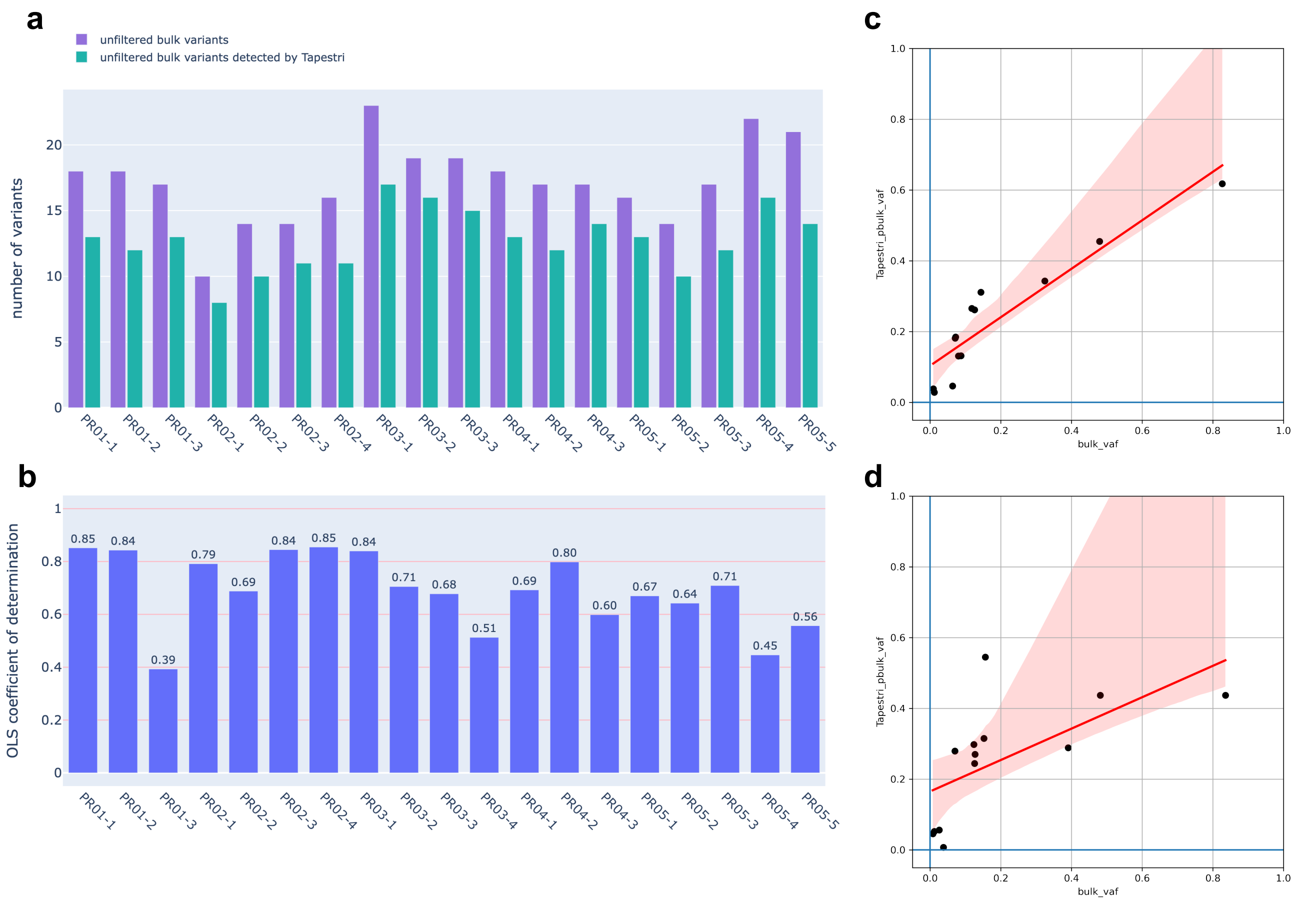

Supplementary figure 4: Bulk vs snDNA-seq variant comparison

a. For 18 samples of the same bulk WES sequencing cohort, histogram showing the number of unfiltered (**Methods**) variants called by bulk (purple) and among them, the number detected by snDNA-seq (green).
b. Per-sample linear regression results of bulk VAF vs snDNA-seq pseudobulk VAF for all shared variants, except for those that have less than or equal to 2 alternative reads in bulk results; coefficient of determination (R2) is plotted on the y axis.
c-d. Representative linear correlation of bulk VAF vs snDNA-seq pseudobulk VAF for shared variants of sample PR01-1 (R2 = 0.85), PR01-3 (R2 = 0.39). Area shaded represents confidence interval = 90% around the regression line.
Source data are provided as a Source Data file.

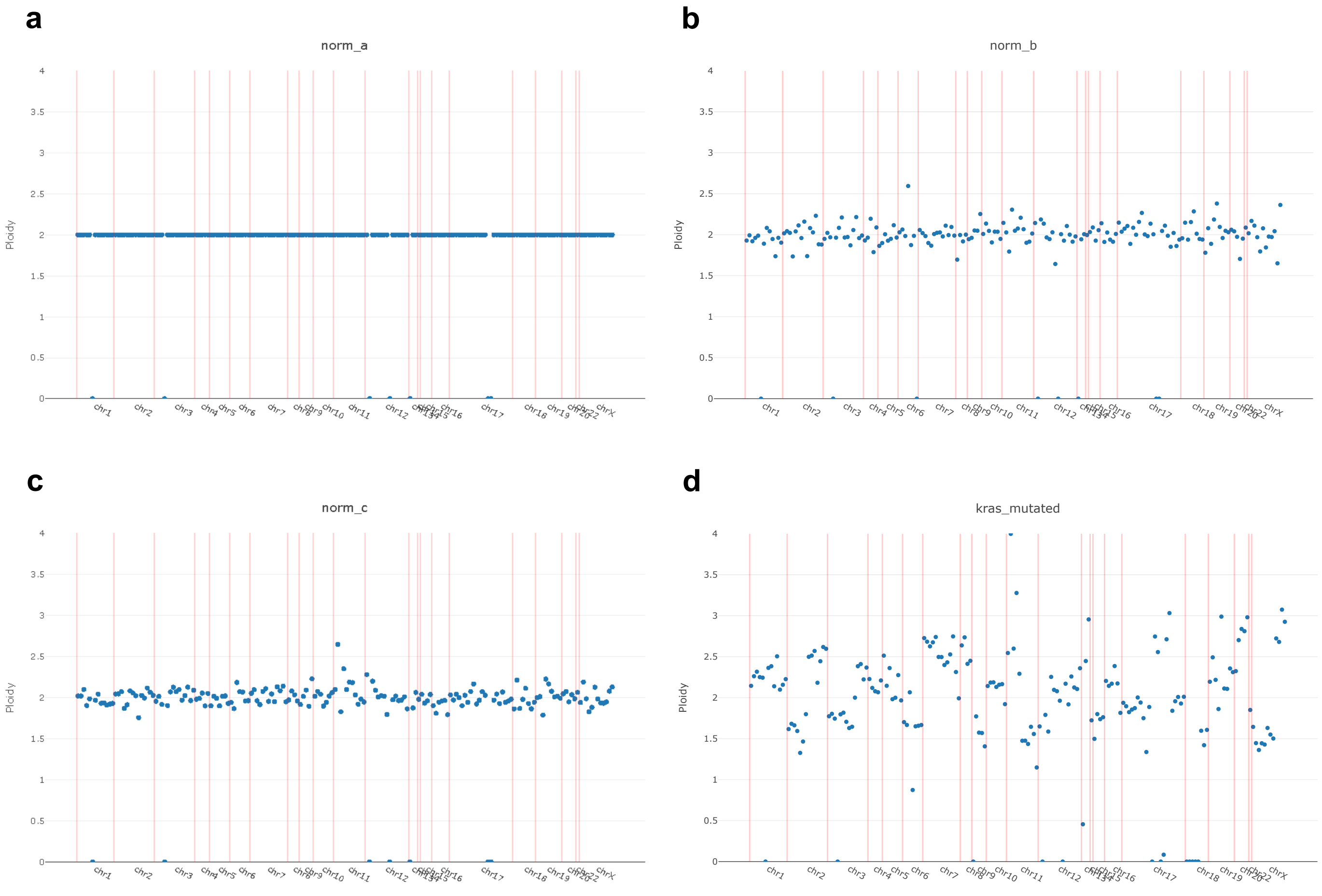

Supplementary figure 5: Clone-median per-amplicon ploidy for sample PA02-1.

Ploidy was calculated as follows:
(1) Read count per (cell * amplicon) is normalized both across cells and amplicons as described in Methods.
(2) KRAS-mutated group is identified as cells carrying KRAS HOM/HET genotype; all other cells are assigned as putative normal cells.
(3) Putative normal cells are randomly split into 3 equally sized groups norm_a, norm_b, norm_c. Norm_a is used as diploid baseline and all other groups' absolute ploidy is calculated as the ratio of their normalized read counts to norm_a' normalized read counts. For sample PA02-1, the resulting absolute per-amplicon ploidy for each group is plotted above (**a-d**).
Source data are provided as a Source Data file.

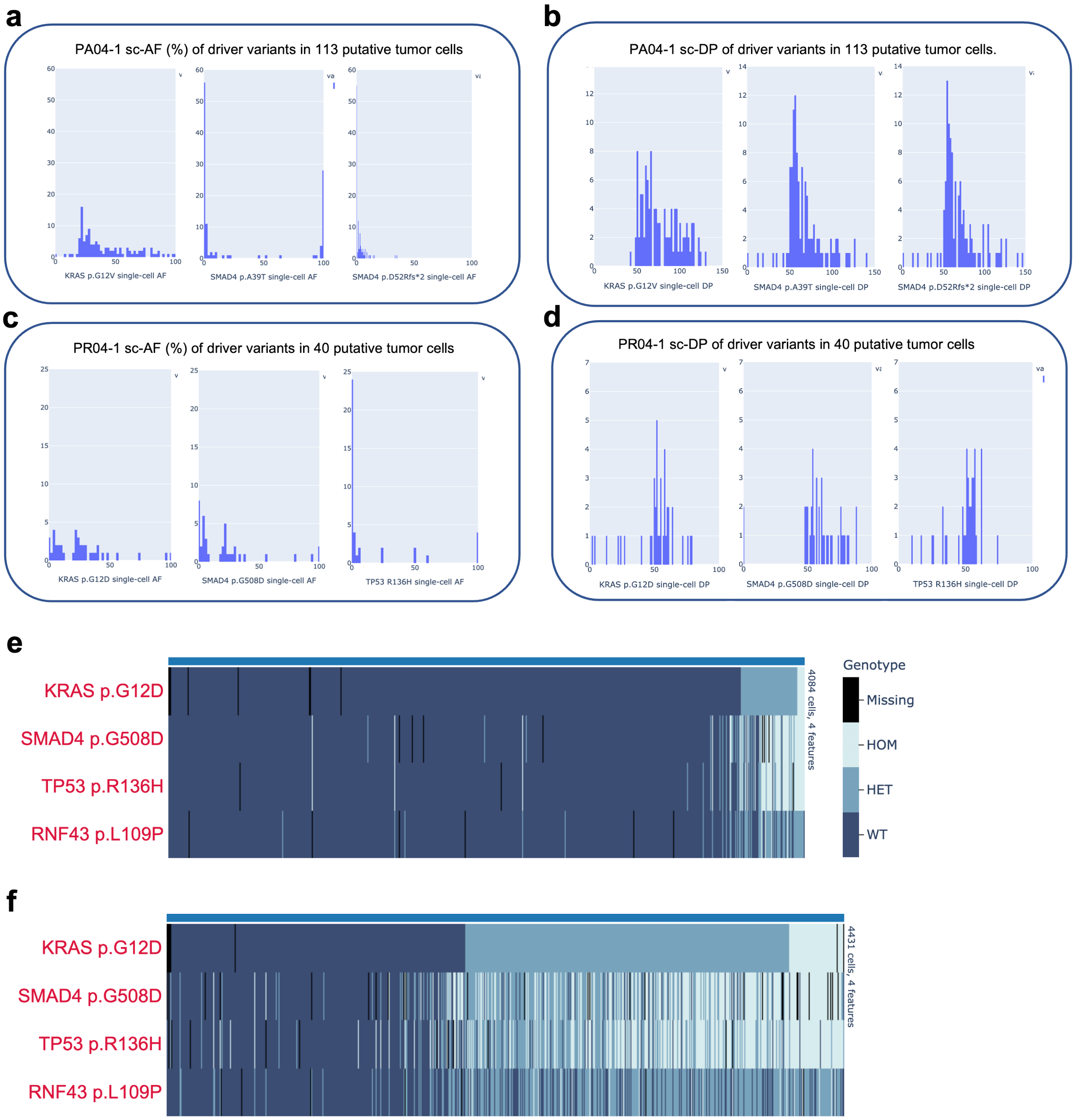

Supplementary figure 6: Tapestri snDNA-seq's performance in limiting settings

a-b. Distribution of single-cell allele frequency (AF, **a**), read depth (DP, **b**) of the 3 main driver variants of sample PA04-1 in the 113 putative tumor cells out of 479 total cells.
c-d. Distribution of single-cell AF (**c**), DP(**d**) of the 3 main driver variants of sample PR04-1 in the 40 putative tumor cells.
e-f. single-cell genotype heatmap of samples PR04-2 (**e**), PR04-3 (**f**), which are two other samples from the same tumor as PR04-1. Cells are again sorted based on KRAS VAF in ascending order from left to right.
Source data are provided as a Source Data file.

a

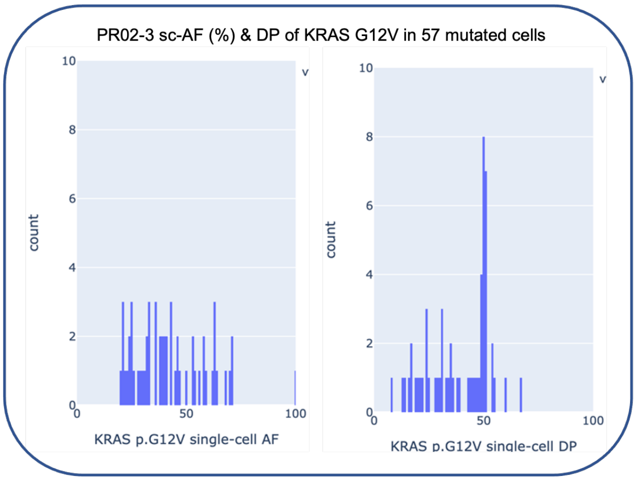

b

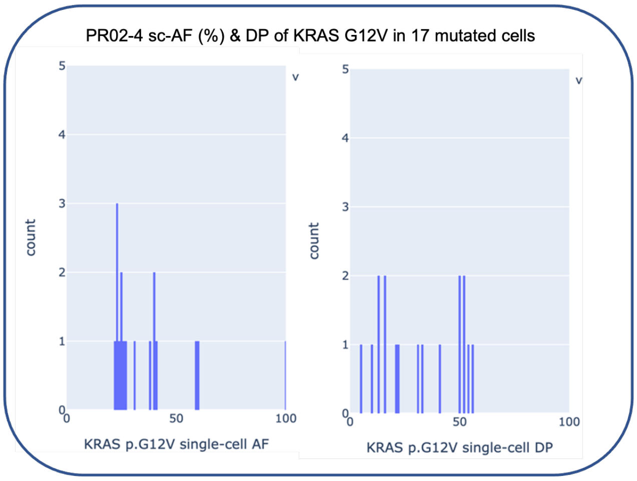

c

Digital Droplet PCR			
**Assay**	**Sample ID**	**Droplet count mutated**	**Droplet count wildtype**
KRAS p.G12D	PR02-1	401	1765
KRAS p.G12D	PR02-3	1048	3608
KRAS p.G12D	Water	0	0
KRAS p.G12D	gDNA WT control	0	2283
KRAS p.G12D	Mutated positive control	2655	2513
KRAS p.G12V	PR02-1	0	1821
KRAS p.G12V	PR02-3	171	3377
KRAS p.G12V	Water	0	0
KRAS p.G12V	gDNA WT control	0	1894
KRAS p.G12V	Mutated positive control	748	1910

Supplementary Figure 7: snDNA-seq identified a minor KRAS p.G12V mutation mutually exclusive with a major KRAS p.G12D mutation in the same tumor

a-b. Distribution of single-cell allele frequency (AF, left), read depth (DP, right) of KRAS p.G12V mutation in cells where it was mutated in sample PR02-3 (**a**), PR02-4 (**b**).
c. Digital droplet PCR results on KRAS variants in samples PR02-1 (used instead of PR02-4 because the latter's nuclei material was depleted) and PR02-3's leftover nuclei from Tapestri runs.
Source data are provided as a Source Data file.

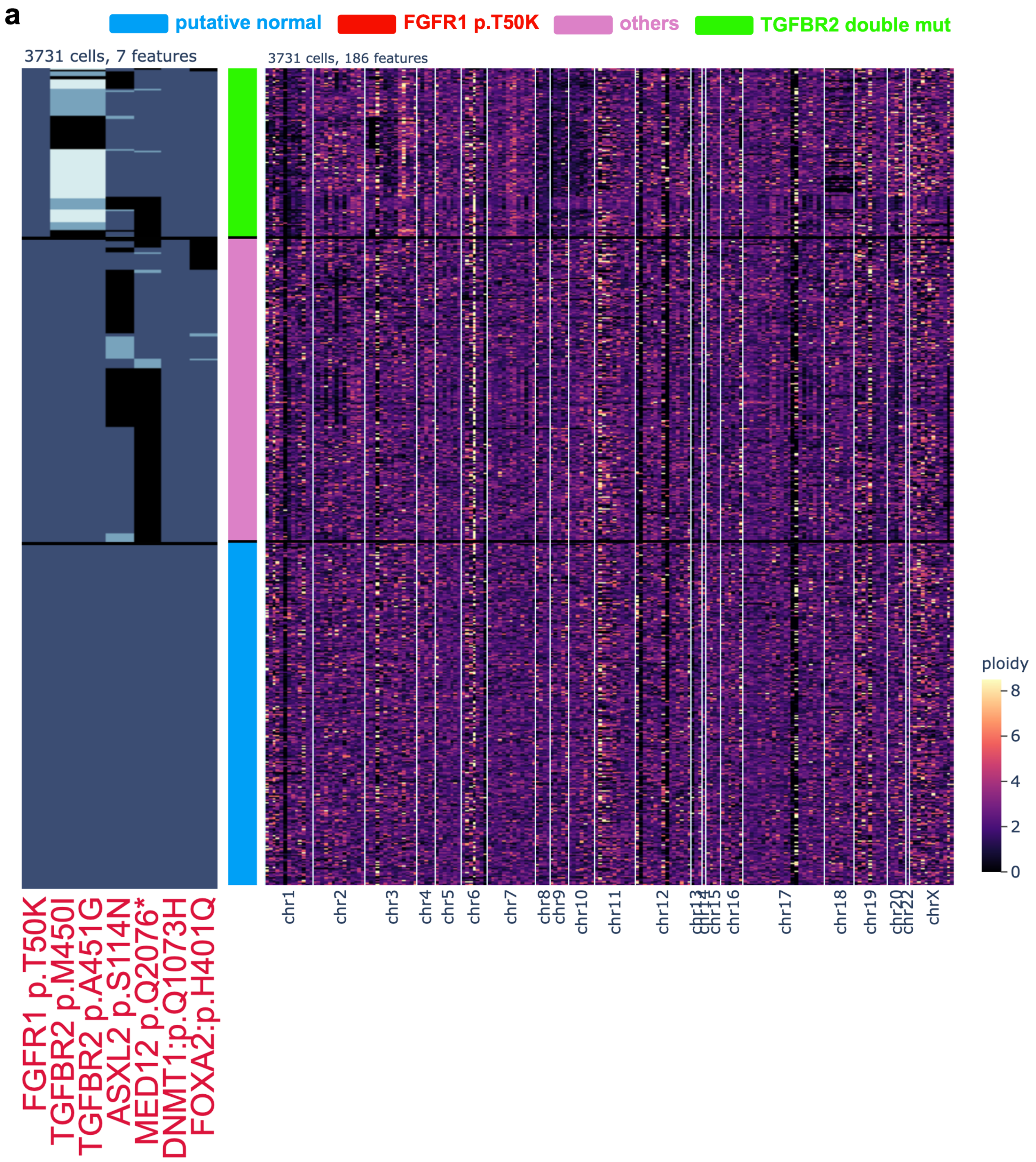

Supplementary Figure 8: single-cell SNV and CNV results of a KRAS WT PDAC

a-c. Single-cell genotype heatmap of 7 important genomic variants pre-identified by bulk WES (left) and genome-wide per-amplicon ploidy heatmap (right) for samples PR01-1 (**a**), PR01-2 (**b**), PR01-3 (**c**). Each cell's clone identity (middle) is colored as shown by labels. The FGFR1 and TGFBR2 SNV clones are defined as cells with non-WT genotype of each gene. The "putative normal" clone is defined as cells with "WT" genotype of all 7 genetic variants. Cells are hierarchically clustered within each clone.

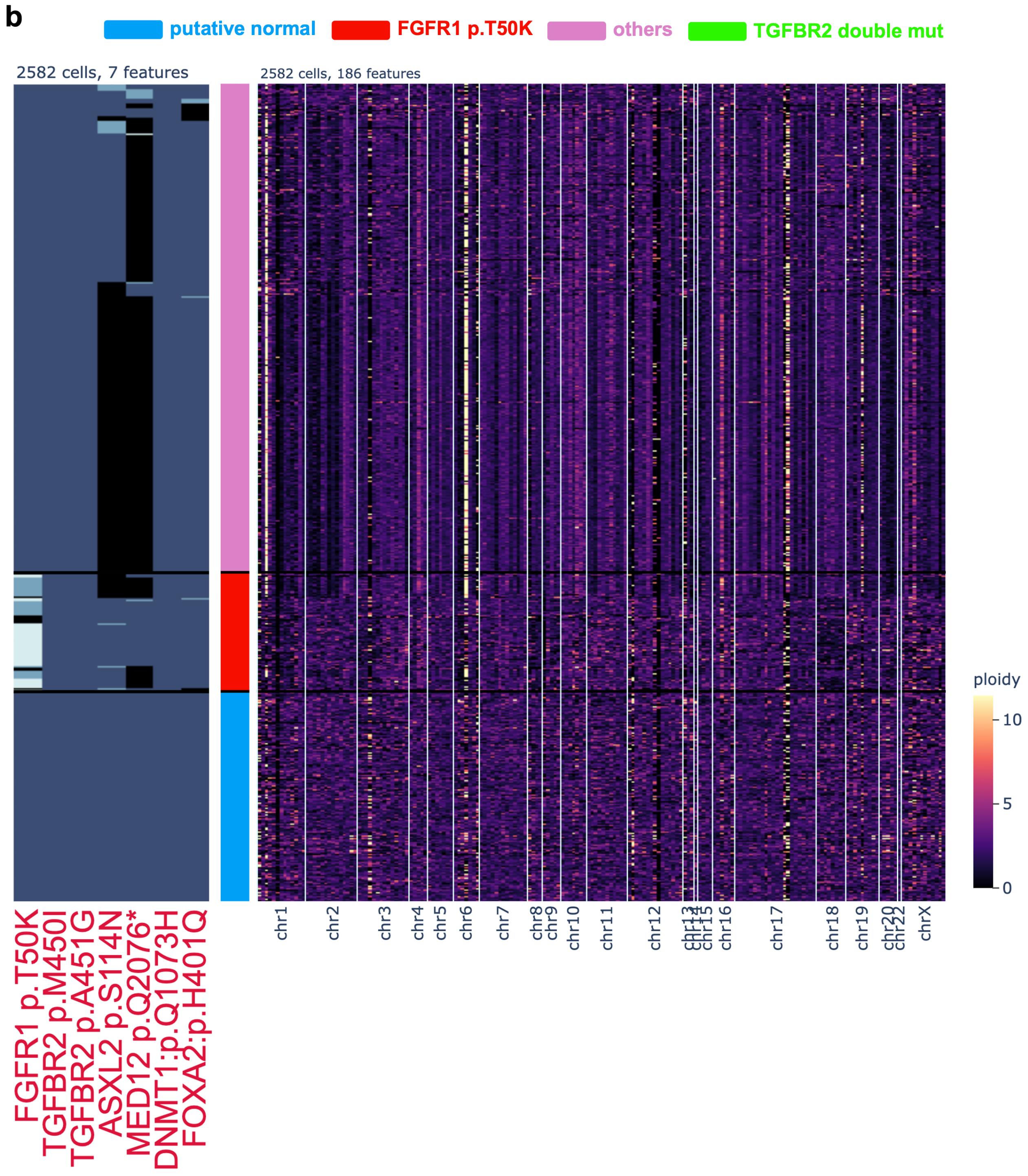

Supplementary Figure 8: single-cell SNV and CNV results of a KRAS WT PDAC (CONTINUED)

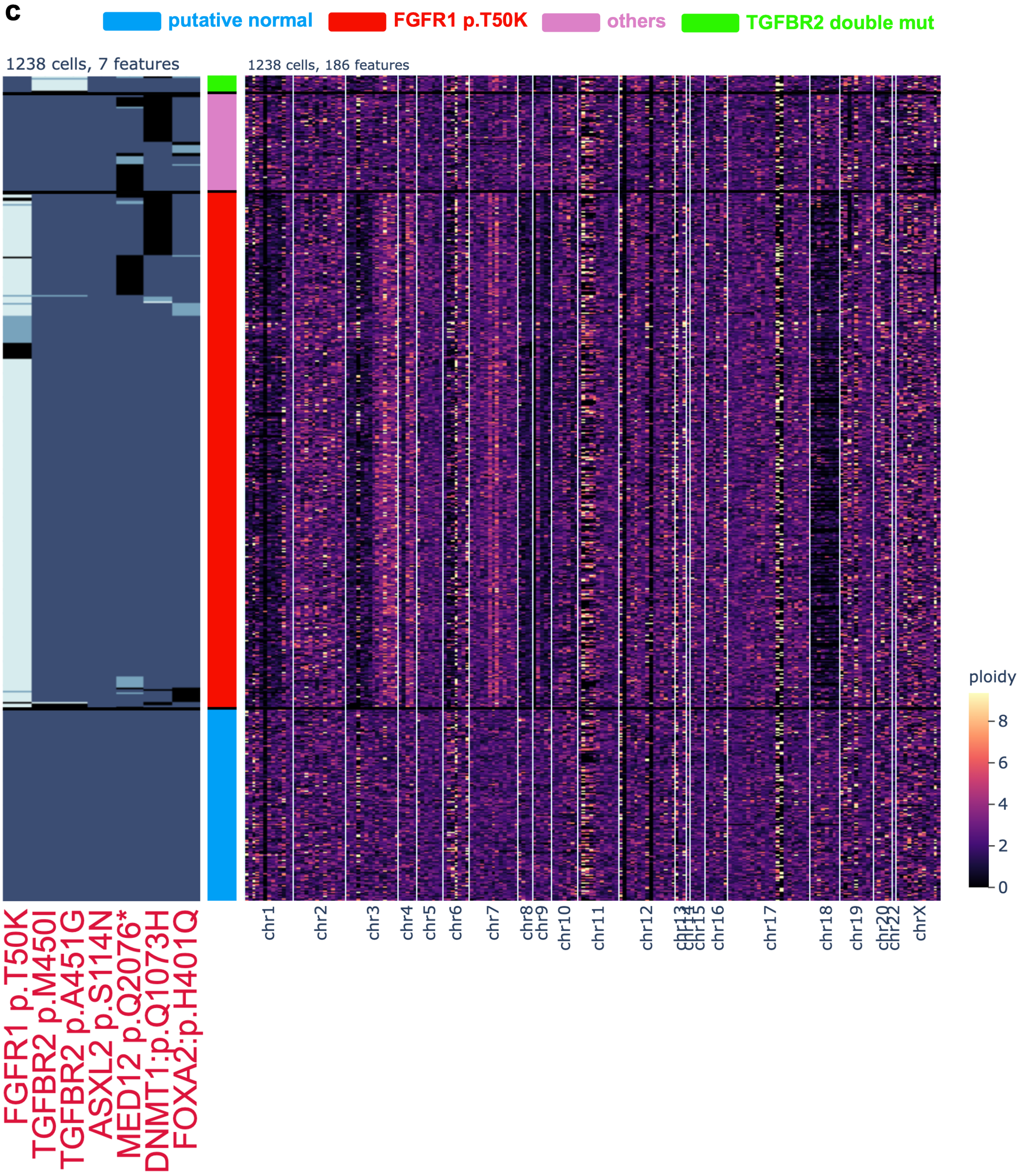

Supplementary Figure 8: single-cell SNV and CNV results of a KRAS WT PDAC (CONTINUED)

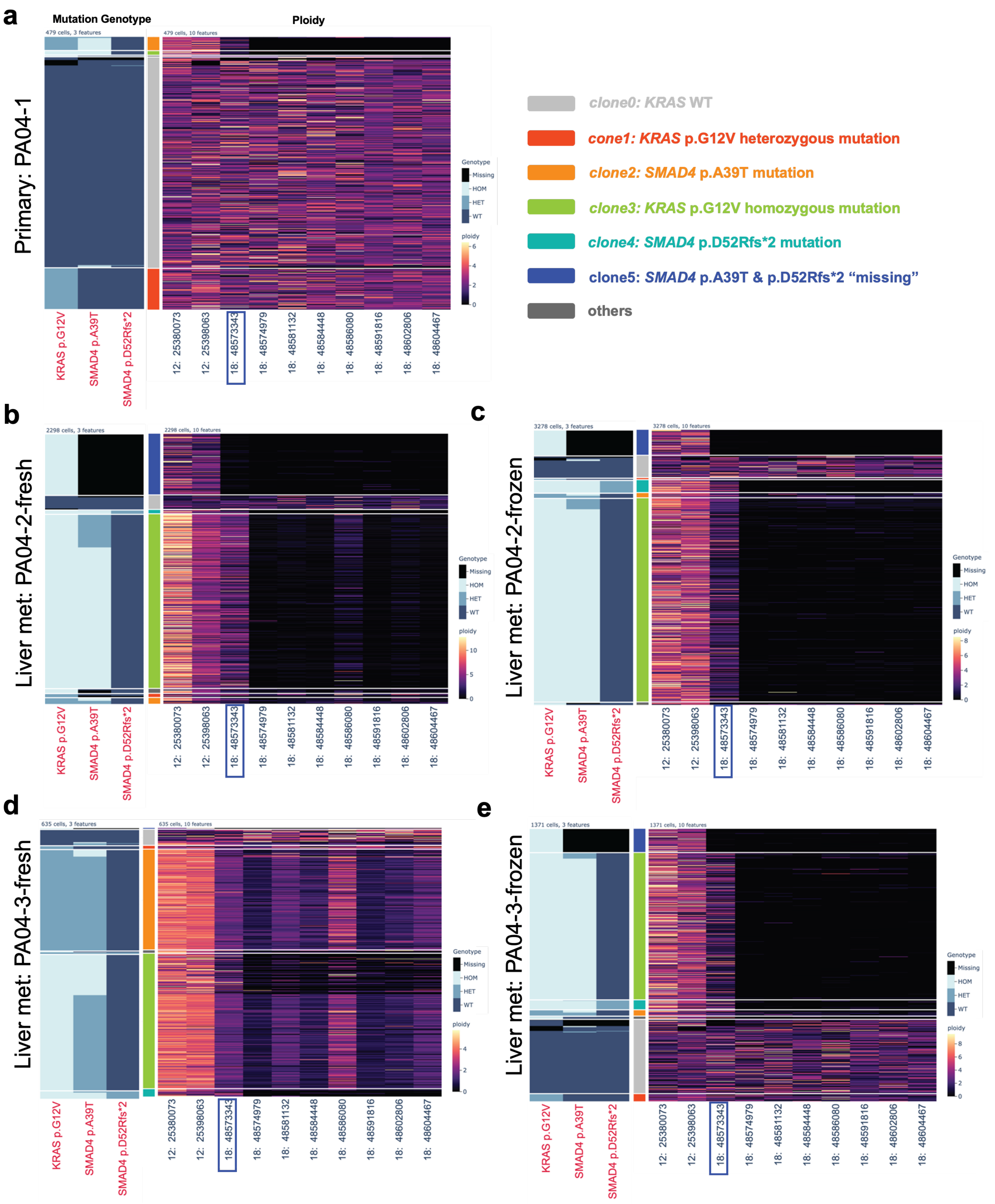

Supplementary Figure 9: raw SNV and CNV data of case PA04

a-e. Combined genotype and ploidy analysis of 3 multiregional samples of PDAC autopsy case PA04. In each panel:

(i) left: single-cell genotype heatmap (of important KRAS and SMAD4 variants preidentified by bulk WGS) is placed on the left.
(ii) middle: each cell's clone identity, which is manually assigned based on KRAS and SMAD4 mutated loci's genotype as shown in the legend.
(iii) single-cell per-amplicon ploidy heatmap. Only amplicons targeting KRAS and SMAD4 are included. Each amplicon's starting genomic location is labeled on the x-axis. The amplicon where the SMAD4 p.A39T and p.D52Rfs*2 mutations took place is outlined in blue.

Cells (rows) are sorted hierarchically within each clone. Each panel corresponds to PA04-1 (**a**, primary tumor), PA04-2-fresh (**b**, liver met slice 1, fresh nuclei), PA04-2-frozen (**c**, liver met slice 1, frozen nuclei), PA04-3-fresh (**d**, liver met slice 2, fresh nuclei) and PA04-3-frozen (**e**, liver met slice 2, frozen nuclei).

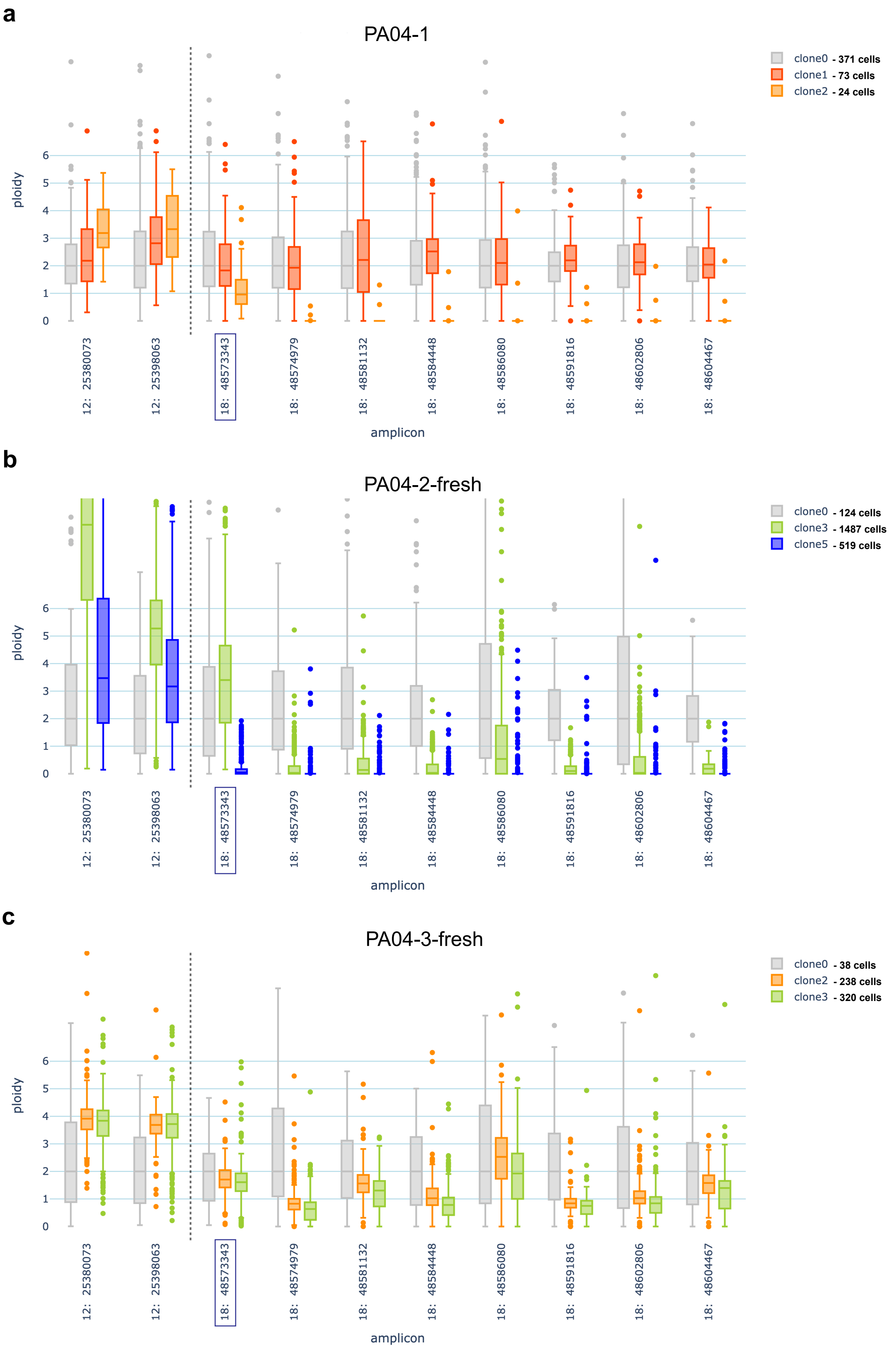

Supplementary Figure 10: clone-level per-amplicon ploidy for KRAS and SMAD4 in case PA04

a-c. Boxplot showing distribution of single cell ploidy for KRAS, SMAD4 in sample PA04-1 (a), PA04-2-fresh (b), PA04-3-fresh (c), split by clone identities as defined in Supplementary Figure 9. Each box spans from quartile 1 (Q1) to quartile 3 (Q3). The second quartile (Q2) is marked by a line inside the box. The whiskers correspond to the box' edges +/- 1.5 times the interquartile range (IQR: Q3-Q1). Only sample points lying outside the whiskers are shown. Sample points with ploidy > 10 are clipped from the images. Each amplicon's starting genomic location is labeled on the x-axis and a dashed line is drawn to separate the two different genes. The amplicon where the SMAD4 p.A39T and p.D52Rfs*2 mutations took place is outlined in blue.
Source data are provided as a Source Data file.

Supplementary Table 1: sample sheet

Background information					Nuclei extraction technical information				Tapestri pipeline run metadata				
case code	sample type	sample code	sample anatomical site	matched bulk data	tumor purity (pathologist)[1]	tumor purity (sc-seq result)	nuclei conc. before encapsulation (/ul)	final Tapestri yield (#nuclei)	number of total read pairs (million)	% reads mapped to panel genomic region	total number of barcodes sequenced	% DNA read pairs assigned to cells	read depth (mean reads/cell/amplicon)
PA01	autopsy	PA01-1	pancreas primary	WES	low	medium	4000	3151	202.28	88.23	1.57	46.08	159
PA01	autopsy	PA01-2	pancreas primary	WES	low	medium	900	1269	256.41	77.23	1.55	53.64	582
PA02	autopsy	PA02-1-sorted	liver met	Sanger sequencing	medium	medium	4000	2501	164.82	93.41	1.48	55.53	196
PA02	autopsy	PA02-1-unsorted	liver met	Sanger sequencing	medium	medium	4000	1253	213.87	95.06	1.55	45.4	416
PA03	autopsy	PA03-1	liver met	WES	N/A[3]	medium	N/A[3]	782	148.53	94.62	1.09	48.97	500
PA03	autopsy	PA03-2	pancreas primary	WES	low	low	3200	5694	149.18	88.78	1.37	63.78	89
PA04	autopsy	PA04-1	pancreas primary	WGS	medium	medium	600	479	142.39	93.87	1.35	70.4	1125
PA04	autopsy	PA04-2-fresh	liver met	WGS (different slice)	high	high	3000	2298	182.4	86.33	1.67	20.47	87
PA04	autopsy	PA04-2-frozen	liver met	WGS (different slice)	high	high	4000	2196	277.76	90.35	1.67	37.12	169
PA04	autopsy	PA04-3-fresh	liver met	WGS (different slice)	medium	high	4000	635	309.6	92.1	1.72	20.21	529
PA04	autopsy	PA04-3-frozen	liver met	WGS (different slice)	medium	high	3500	1371	258.98	53.42	1.45	16.83	170
PA05	autopsy	PA05-1-A	pancreas primary	WGS (different slice)	medium	low	2000	4249	169.27	89.84	1.6	68.37	146
PA05	autopsy	PA05-1-B	pancreas primary	WGS (different slice)	medium	low	4200	2244	151.06	90.11	1.29	49.75	180
PO01	organoid	PO01-1	pancreas biopsy	WES	high[2]	high	2600	2374	191.54	88.25	1.71	30.65	132
PR01	resection	PR01-1	pancreas primary	MSK-IMPACT, WES	medium	low	4000	3731	131.54	87.79	1.51	51.27	97
PR01	resection	PR01-2	pancreas primary	MSK-IMPACT, WES	medium	low	2500	2582	108.07	88.79	1.35	35.55	79
PR01	resection	PR01-3	pancreas primary	MSK-IMPACT, WES	low	medium	4000	1238	128.69	85.84	1.26	59.84	334
PR02	resection	PR02-1	pancreas primary	MSK-IMPACT, WES	low	low	3800	1471	145.3	86.83	1.16	58.13	308
PR02	resection	PR02-2	pancreas primary	MSK-IMPACT, WES	low	low	4000	780	136.44	80.75	1.13	56.42	530
PR02	resection	PR02-3	pancreas primary	MSK-IMPACT, WES	medium	medium	4000	3545	69.85	92	1.09	52.83	55
PR02	resection	PR02-4	pancreas primary	MSK-IMPACT, WES	low	low	2000	3609	52.35	89.03	0.99	51.01	39
PR03	resection	PR03-1	pancreas primary	MSK-IMPACT, WES	low	medium	4000	3617	119.29	90.19	1.47	36.15	64
PR03	resection	PR03-2	pancreas primary	MSK-IMPACT, WES	low	medium	3500	1552	143.58	81.58	1.29	35.4	176
PR03	resection	PR03-3	pancreas primary	MSK-IMPACT, WES	low	medium	4000	3278	300.83	91.65	1.8	55.26	406
PR03	resection	PR03-4	pancreas primary	MSK-IMPACT, WES	medium	medium	3200	2515	141.2	91.11	1.53	57.56	173
PR04	resection	PR04-1	pancreas primary	MSK-IMPACT, WES	medium	low	3000	3866	146.2	91.74	1.55	51.52	104
PR04	resection	PR04-2	pancreas primary	MSK-IMPACT, WES	low	low	3500	4084	137.89	89.88	1.51	62.49	113
PR04	resection	PR04-3	pancreas primary	MSK-IMPACT, WES	low	medium	4400	4431	278.54	92.79	1.7	53.94	182
PR05	resection	PR05-1	pancreas primary	MSK-IMPACT, WES	medium	high	4000	7558	154.3	91.9	1.66	48.62	53
PR05	resection	PR05-2	pancreas primary	MSK-IMPACT, WES	medium	high	4000	4638	143.74	91.47	1.54	43.87	73
PR05	resection	PR05-3	pancreas primary	MSK-IMPACT, WES	medium	medium	4000	3651	341.21	92.19	1.79	68.12	342
PR05	resection	PR05-4	pancreas primary	MSK-IMPACT, WES	low	low	3500	2397	122.39	90.38	1.35	59.61	163
PR05	resection	PR05-5	pancreas primary	MSK-IMPACT, WES	low	medium	4000	1409	161.88	89.65	1.5	51.21	316
PR06	resection	PR06-1	pancreas primary	WES on matched organoid	low	medium	4000	7282	222.68	91.91	1.81	39.13	64
PR07	resection	PR07-1	pancreas primary	WES on matched organoid	medium	low	2600	4166	145.44	88.76	1.42	59.87	112
PR08	resection	PR08-1	pancreas primary	WES on matched organoid	medium	medium	2600	2063	202.96	90.42	1.49	46.99	248
PR09	resection	PR09-1	pancreas primary	WES on matched organoid	medium	medium	2000	1767	276.5	88.21	1.71	53.2	447
PR10	resection	PR10-1	pancreas primary	WES on matched organoid	medium	medium	1800	3220	117.34	89.63	1.28	63	123
MixA	resection	PR03-3 & PR05-4	pancreas primary	MSK-IMPACT, WES	N/A	N/A	3500	1064	200.05	49.14	1.51	30.97	313
MixB	resection	PR03-3 & PR05-4	pancreas primary	MSK-IMPACT, WES	N/A	N/A	3500	1931	401.28	51.38	1.57	32.06	358

Notes:
1. low purity: 25%; medium: 25-75%; high: >75%
2. organoid was assumed to be 100% tumor
3. data was not collected

Supplementary Table 2- Tapestri panel coverage of canonical exons

amplicon_chr	insert_start	insert_end	amplicon_number	exon_chr	exon_start	exon_end	gene_name	canonical_transcript	exon_number	strand	#bp_overlap
chr1	27057559	27057771	AMPL50980	chr1	27057640	27058097	ARID1A	uc001bmv.1	2	+	131
chr1	27087373	27087587	AMPL70456	chr1	27087344	27087589	ARID1A	uc001bmv.1	4	+	214
chr1	27092546	27092742	AMPL87242	chr1	27092709	27092859	ARID1A	uc001bmv.1	8	+	33
chr1	27097525	27097699	AMPL87243	chr1	27097607	27097819	ARID1A	uc001bmv.1	11	+	92
chr1	27099426	27099592	AMPL87244	chr1	27099300	27099480	ARID1A	uc001bmv.1	13	+	54
chr1	27099747	27099947	AMPL41862	chr1	27099834	27099989	ARID1A	uc001bmv.1	14	+	113
chr1	27100765	27100976	AMPL87246	chr1	27100817	27101713	ARID1A	uc001bmv.1	17	+	159
chr1	27105718	27105932	AMPL87247	chr1	27105511	27108603	ARID1A	uc001bmv.1	19	+	214
chr1	27106587	27106734	AMPL87248	chr1	27105511	27108603	ARID1A	uc001bmv.1	19	+	147
chr1	27106950	27107136	AMPL87249	chr1	27105511	27108603	ARID1A	uc001bmv.1	19	+	186
chr1	45292813	45292985	AMPL87250	chr1	45292836	45292983	PTCH2	uc010olf.2	6	-	147
chr1	65338840	65339055	AMPL81995	chr1	65339050	65339208	JAK1	uc001dbu.1	20	-	5
chr1	120458813	120458992	AMPL87252	chr1	120454173	120459319	NOTCH2	uc001eik.3	0	-	179
chr10	55568774	55568980	AMPL87322	.	-1	-1	.	.	.	.	0
chr10	55581521	55581729	AMPL87323	chr10	55580857	55583120	PCDH15	uc010qhy.1	0	-	208
chr10	55826408	55826551	AMPL87324	chr10	55826514	55826647	PCDH15	uc010qhy.1	16	-	37
chr10	56128747	56128951	AMPL87325	chr10	56128877	56129037	PCDH15	uc010qhy.1	29	-	74
chr10	63759883	63760095	AMPL70484	chr10	63759847	63760082	ARID5B	uc001jlt.2	3	+	199
chr10	89717479	89717672	AMPL56571	chr10	89717607	89717778	PTEN	uc001kfb.3	6	+	65
chr10	89720561	89720732	AMPL70428	chr10	89720648	89720877	PTEN	uc001kfb.3	7	+	84
chr11	57576696	57576892	AMPL87329	chr11	57576743	57576940	CTNND1	uc001nmc.4	14	+	149
chr11	64572398	64572589	AMPL84848	chr11	64572503	64572672	MEN1	uc001obn.3	1	-	86
chr11	64573536	64573750	AMPL87331	chr11	64573701	64573842	MEN1	uc001obn.3	3	-	49
chr11	64574358	64574572	AMPL87332	chr11	64574480	64574572	MEN1	uc001obn.3	4	-	92
chr11	64577118	64577334	AMPL79919	chr11	64577119	64577606	MEN1	uc001obn.3	8	-	215
chr11	108123364	108123540	AMPL70485	.	-1	-1	.	.	.	.	0
chr11	108138971	108139130	AMPL70486	.	-1	-1	.	.	.	.	0
chr11	108153437	108153642	AMPL70487	chr11	108153434	108153608	ATM	uc001pkb.1	24	+	171
chr11	108191986	108192156	AMPL44533	chr11	108192025	108192149	ATM	uc001pkb.1	44	+	124
chr11	108235881	108236095	AMPL52758	chr11	108235806	108235947	ATM	uc001pkb.1	61	+	66
chr11	108235881	108236095	AMPL52758	chr11	108236049	108239828	ATM	uc001pkb.1	62	+	46
chr11	125502860	125503070	AMPL70490	chr11	125503055	125503248	CHEK1	uc001qcg.4	5	+	15
chr12	18793284	18793497	AMPL70366	chr12	18793360	18793490	PIK3C2G	uc001rdt.3	30	+	130

chr12	18800822	18800948	AMPL70367	chr12	18800807	18801354	PIK3C2G	uc001rdt.3		31	+	126
chr12	25380105	25380275	AMPL52906	chr12	25380165	25380348	KRAS	uc001rgp.1		3	-	110
chr12	25398087	25398284	AMPL41099	chr12	25398205	25398331	KRAS	uc001rgp.1		4	-	79
chr12	25398087	25398284	AMPL41099	chr12	25398272	25398299	DD157417	uc021qwd.1		0	+	12
chr12	46231233	46231411	AMPL87344	chr12	46231278	46231492	ARID2	uc001ros.1		9	+	133
chr12	46244510	46244722	AMPL87345	chr12	46243816	46246681	ARID2	uc001ros.1		14	+	212
chr12	49415683	49415899	AMPL87346	chr12	49415823	49415936	KMT2D	uc001rta.4		1	-	76
chr12	49423980	49424194	AMPL87347	chr12	49424060	49424224	KMT2D	uc001rta.4		12	-	134
chr12	49434102	49434294	AMPL56594	chr12	49433504	49435320	KMT2D	uc001rta.4		23	-	192
chr12	49439992	49440207	AMPL82073	chr12	49440040	49440209	KMT2D	uc001rta.4		38	-	167
chr12	49444560	49444777	AMPL42560	chr12	49443462	49444575	KMT2D	uc001rta.4		43	-	15
chr12	49444560	49444777	AMPL42560	chr12	49444666	49446209	KMT2D	uc001rta.4		44	-	111
chr12	52378822	52379019	AMPL87351	chr12	52378973	52379134	ACVR1B	uc010snn.2		6	+	46
chr12	56481476	56481675	AMPL87352	chr12	56481576	56481699	ERBB3	uc001sjh.3		5	+	99
chr12	133225672	133225893	AMPL87353	chr12	133225889	133226103	POLE	uc001uks.1		18	-	4
chr12	133237565	133237762	AMPL59905	chr12	133237552	133237752	POLE	uc001uks.1		24	-	187
chr13	32913227	32913426	AMPL87355	chr13	32910399	32915335	BRCA2	uc001uub.1		10	+	199
chr13	32928990	32929165	AMPL87356	chr13	32928995	32929427	BRCA2	uc001uub.1		13	+	170
chr13	41134675	41134889	AMPL70371	chr13	41133643	41134999	FOXO1	uc001uxl.4		1	-	214
chr14	71199881	71200038	AMPL87358	chr14	71199253	71200061	MAP3K9	uc001xml.3		1	-	157
chr15	41990855	41991053	AMPL70437	.	-1	-1	.	.	.		.	0
chr15	67473512	67473729	AMPL70372	chr15	67473576	67473793	SMAD3	uc002aqj.3		5	+	153
chr15	67479593	67479810	AMPL70373	chr15	67479700	67479849	SMAD3	uc002aqj.3		7	+	110
chr15	88472435	88472632	AMPL87362	chr15	88472419	88472667	NTRK3	uc002bme.2		3	-	197
chr16	346978	347185	AMPL87363	chr16	347053	347228	AXIN1	uc002cgp.2		4	-	132
chr16	23646612	23646823	AMPL87364	chr16	23646180	23647657	PALB2	uc002dlx.1		9	-	211
chr16	65038556	65038772	AMPL84944	chr16	65038542	65038946	CDH11	uc002eoi.3		10	-	216
chr16	68849409	68849577	AMPL87366	chr16	68849415	68849664	CDH1	uc002ewg.1		9	+	162
chr16	68857183	68857357	AMPL87367	chr16	68857299	68857531	CDH1	uc002ewg.1		12	+	58
chr16	89858670	89858883	AMPL70497	chr16	89858876	89858957	FANCA	uc002fou.1		31	-	7
chr17	7572773	7572991	AMPL40059	chr17	7571717	7573010	TP53	uc002gij.3		0	-	218
chr17	7572773	7572991	AMPL40059	chr17	7572989	7573013	HV941431	uc031qyr.1		0	+	2
chr17	7573829	7574039	AMPL55785	chr17	7573924	7574035	TP53	uc002gij.3		1	-	111
chr17	7576902	7577121	AMPL45911	chr17	7576850	7576928	TP53	uc002gij.3		2	-	26
chr17	7576902	7577121	AMPL45911	chr17	7577016	7577157	TP53	uc002gij.3		3	-	105
chr17	7576902	7577121	AMPL45911	chr17	7577060	7577085	HV941428	uc031qys.1		0	+	25
chr17	7577388	7577601	AMPL87372	chr17	7577496	7577524	HV941486	uc021tpf.1		0	+	28
chr17	7577388	7577601	AMPL87372	chr17	7577496	7577610	TP53	uc002gij.3		4	-	105
chr17	7577388	7577601	AMPL87372	chr17	7577555	7577592	HV941429	uc021tpg.1		0	+	37
chr17	7578302	7578517	AMPL41629	chr17	7578368	7578556	TP53	uc002gij.3		6	-	149

chr17	7578302	7578517	AMPL41629	chr17	7578431	7578456	HV941442	uc031qyv.1	0	+	25
chr17	7579315	7579509	AMPL81699	chr17	7579309	7579339	HV941444	uc031qyw.1	0	+	24
chr17	7579315	7579509	AMPL81699	chr17	7579309	7579592	TP53	uc002gij.3	7	-	194
chr17	7579551	7579712	AMPL81700	chr17	7579309	7579592	TP53	uc002gij.3	7	-	41
chr17	7579551	7579712	AMPL81700	chr17	7579697	7579723	TP53	uc002gij.3	8	-	15
chr17	11984562	11984778	AMPL87376	chr17	11984670	11984849	MAP2K4	uc002gnj.3	2	+	108
chr17	11998744	11998949	AMPL87377	chr17	11998889	11999013	MAP2K4	uc002gnj.3	3	+	60
chr17	12016447	12016575	AMPL70382	chr17	12016547	12016679	MAP2K4	uc002gnj.3	6	+	28
chr17	12032328	12032543	AMPL87379	chr17	12032453	12032606	MAP2K4	uc002gnj.3	8	+	90
chr17	29661735	29661925	AMPL87380	chr17	29661853	29662051	NF1	uc002hgg.3	39	+	72
chr17	29685798	29686011	AMPL87381	chr17	29685984	29686035	NF1	uc002hgg.3	55	+	27
chr17	37881153	37881350	AMPL87382	chr17	37880976	37881166	ERBB2	uc002hso.3	19	+	13
chr17	37881153	37881350	AMPL87382	chr17	37881299	37881459	ERBB2	uc002hso.3	20	+	51
chr17	41244119	41244332	AMPL87383	chr17	41243449	41246879	BRCA1	uc002ict.3	14	-	213
chr17	41245435	41245590	AMPL87384	chr17	41243449	41246879	BRCA1	uc002ict.3	14	-	155
chr17	41245757	41245945	AMPL87385	chr17	41243449	41246879	BRCA1	uc002ict.3	14	-	188
chr17	56434930	56435090	AMPL87386	chr17	56434826	56436186	RNF43	uc002iwh.4	1	-	160
chr17	56435188	56435374	AMPL87387	chr17	56434826	56436186	RNF43	uc002iwh.4	1	-	186
chr17	56439744	56439945	AMPL87388	chr17	56439902	56440011	RNF43	uc002iwh.4	4	-	43
chr17	56440742	56440961	AMPL87389	chr17	56440633	56440769	RNF43	uc002iwh.4	5	-	27
chr17	56440742	56440961	AMPL87389	chr17	56440884	56440963	RNF43	uc002iwh.4	6	-	77
chr17	56448126	56448321	AMPL70385	chr17	56448269	56448396	RNF43	uc002iwh.4	7	-	52
chr18	48573368	48573568	AMPL84306	chr18	48573287	48573667	SMAD4	uc010xdp.2	1	+	200
chr18	48575011	48575209	AMPL87392	chr18	48575053	48575232	SMAD4	uc010xdp.2	2	+	156
chr18	48581164	48581300	AMPL87393	chr18	48581148	48581365	SMAD4	uc010xdp.2	4	+	136
chr18	48584477	48584666	AMPL87394	chr18	48584492	48584616	SMAD4	uc010xdp.2	5	+	124
chr18	48586112	48586263	AMPL87395	chr18	48586233	48586288	SMAD4	uc010xdp.2	7	+	30
chr18	48591840	48592011	AMPL87396	chr18	48591790	48591978	SMAD4	uc010xdp.2	8	+	138
chr18	48602826	48603046	AMPL87397	chr18	48603005	48603148	SMAD4	uc010xdp.2	10	+	41
chr18	48604494	48604701	AMPL70387	chr18	48604623	48611413	SMAD4	uc010xdp.2	11	+	78
chr19	1206956	1207135	AMPL82618	chr19	1205795	1207204	STK11	uc002lrl.1	0	+	179
chr19	1220245	1220464	AMPL44665	chr19	1220369	1220506	STK11	uc002lrl.1	3	+	95
chr19	10252752	10252940	AMPL70390	chr19	10252701	10252898	DNMT1	uc010xlc.2	11	-	146
chr19	10270548	10270755	AMPL70499	chr19	10270515	10270600	DNMT1	uc010xlc.2	25	-	52
chr19	10270548	10270755	AMPL70499	chr19	10270691	10270741	DNMT1	uc010xlc.2	26	-	50
chr19	11096834	11097050	AMPL87403	chr19	11096862	11097271	SMARCA4	uc010dxo.3	3	+	188
chr19	11143894	11144113	AMPL87404	chr19	11143963	11144195	SMARCA4	uc010dxo.3	25	+	150
chr19	17954035	17954219	AMPL50677	chr19	17954186	17954302	JAK3	uc002nhn.4	20	-	33
chr19	47424869	47425020	AMPL87406	chr19	47421930	47425615	ARHGAP35	uc010ekv.3	0	+	151
chr19	47425154	47425365	AMPL87407	chr19	47421930	47425615	ARHGAP35	uc010ekv.3	0	+	211

chr2	25457043	25457255	AMPL41019	chr2	25455827	25457291	DNMT3A	uc002rgc.4	0	-	212
chr2	25463058	25463271	AMPL87254	chr2	25463168	25463321	DNMT3A	uc002rgc.4	4	-	103
chr2	25466953	25467126	AMPL87255	chr2	25467021	25467209	DNMT3A	uc002rgc.4	8	-	105
chr2	25470315	25470521	AMPL87256	chr2	25470457	25470620	DNMT3A	uc002rgc.4	15	-	64
chr2	25976271	25976451	AMPL70461	chr2	25976400	25976510	ASXL2	uc002rgs.2	2	-	51
chr2	25994178	25994391	AMPL70462	chr2	25994306	25994411	ASXL2	uc002rgs.2	7	-	85
chr2	26022113	26022318	AMPL70463	chr2	26022251	26022406	ASXL2	uc002rgs.2	8	-	67
chr2	48028050	48028263	AMPL70405	chr2	48025747	48028296	MSH6	uc002rwd.4	3	+	213
chr2	61760864	61761054	AMPL65019	chr2	61760904	61761040	XPO1	uc002sbj.3	23	-	136
chr2	80084990	80085190	AMPL87262	chr2	80085136	80085307	CTNNA2	uc010ysf.2	3	+	54
chr2	148657126	148657335	AMPL87263	chr2	148657024	148657138	ACVR2A	uc002twh.3	2	+	12
chr2	148657126	148657335	AMPL87263	chr2	148657310	148657469	ACVR2A	uc002twh.3	3	+	25
chr2	148683482	148683690	AMPL40069	chr2	148683597	148683732	ACVR2A	uc002twh.3	9	+	93
chr2	198266465	198266596	AMPL87265	chr2	198266463	198266614	SF3B1	uc002uue.3	9	-	131
chr2	198266664	198266802	AMPL87266	chr2	198266706	198266856	SF3B1	uc002uue.3	10	-	96
chr20	22562487	22562700	AMPL70500	chr20	22561639	22563794	FOXA2	uc002wsm.3	0	-	213
chr20	31021158	31021304	AMPL87409	chr20	31021084	31021722	ASXL1	uc002wxs.3	10	+	146
chr20	31021350	31021498	AMPL70392	chr20	31021084	31021722	ASXL1	uc002wxs.3	10	+	148
chr20	57415757	57415907	AMPL59385	chr20	57414792	57415943	GNAS	uc021wfl.1	0	+	150
chr20	57484215	57484421	AMPL38817	chr20	57484214	57484273	GNAS	uc002xzw.3	6	+	58
chr20	57484215	57484421	AMPL38817	chr20	57484214	57484273	GNAS	uc021wfl.1	6	+	58
chr20	57484215	57484421	AMPL38817	chr20	57484402	57484480	GNAS	uc002xzw.3	7	+	19
chr20	57484215	57484421	AMPL38817	chr20	57484402	57484480	GNAS	uc021wfl.1	7	+	19
chr22	22153221	22153430	AMPL82112	chr22	22153298	22153419	MAPK1	uc002zvn.3	5	-	121
chr3	30686139	30686353	AMPL87267	chr3	30686236	30686409	TGFBR2	uc003cen.3	2	+	117
chr3	30713625	30713784	AMPL87268	chr3	30713127	30713931	TGFBR2	uc003cen.3	4	+	159
chr3	30715574	30715726	AMPL70340	chr3	30715594	30715740	TGFBR2	uc003cen.3	5	+	132
chr3	30729827	30729987	AMPL87270	chr3	30729873	30730005	TGFBR2	uc003cen.3	6	+	114
chr3	41274883	41275096	AMPL87271	chr3	41274829	41274937	CTNNB1	uc003ckr.2	7	+	54
chr3	41274883	41275096	AMPL87271	chr3	41275017	41275360	CTNNB1	uc003ckr.2	8	+	79
chr3	52637364	52637541	AMPL87272	chr3	52637534	52637750	PBRM1	uc003der.2	12	-	7
chr3	52675872	52676073	AMPL70414	chr3	52675967	52676063	PBRM1	uc003der.2	19	-	96
chr3	77645598	77645812	AMPL87274	chr3	77645771	77645903	ROBO2	uc003dpy.4	18	+	41
chr3	78700816	78701024	AMPL87275	chr3	78700879	78701081	ROBO1	uc003dqe.2	12	-	145
chr3	142280105	142280311	AMPL70344	chr3	142280082	142280265	ATR	uc003eux.4	42	-	160
chr3	169997892	169998090	AMPL70415	chr3	169998012	169998193	PRKCI	uc003fgs.2	8	+	78
chr3	178935938	178936116	AMPL40482	chr3	178935995	178936124	PIK3CA	uc003fjk.3	9	+	121
chr3	178951802	178951995	AMPL87279	chr3	178951879	178952499	PIK3CA	uc003fjk.3	20	+	116
chr3	189612086	189612303	AMPL70345	chr3	189611992	189615070	TP63	uc003fry.2	13	+	217
chr4	55980096	55980303	AMPL70347	chr4	55980290	55980434	KDR	uc003has.3	24	-	13

chr4	79188225	79188416	AMPL87282	chr4	79188392	79188588	FRAS1	uc003hlb.2	8	+		24
chr4	153246964	153247168	AMPL87283	chr4	153247155	153247385	FBXW7	uc003ims.3	2	-		13
chr4	153249293	153249505	AMPL79867	chr4	153249357	153249543	FBXW7	uc003ims.3	3	-		148
chr4	187538940	187539155	AMPL87285	chr4	187538859	187542931	FAT1	uc003izf.3	17	-		215
chr5	11082714	11082931	AMPL87286	chr5	11082805	11082960	CTNND2	uc003jfa.1	6	-		126
chr5	11346393	11346611	AMPL87287	chr5	11346481	11346741	CTNND2	uc003jfa.1	13	-		130
chr5	38949958	38950168	AMPL87288	chr5	38949811	38950824	RICTOR	uc003jlp.2	7	-		210
chr5	38978600	38978768	AMPL70348	chr5	38978682	38978754	RICTOR	uc003jlp.2	29	-		72
chr5	112116288	112116500	AMPL70472	chr5	112116484	112116602	APC	uc003kpy.4	5	+		16
chr5	112157486	112157674	AMPL70473	chr5	112157590	112157690	APC	uc003kpy.4	10	+		84
chr5	112170540	112170760	AMPL70474	chr5	112170645	112170864	APC	uc003kpy.4	14	+		115
chr6	7229232	7229436	AMPL87293	chr6	7229227	7232142	RREB1	uc003mxb.3	9	+		204
chr6	7229498	7229654	AMPL87294	chr6	7229227	7232142	RREB1	uc003mxb.3	9	+		156
chr6	7230435	7230606	AMPL87295	chr6	7229227	7232142	RREB1	uc003mxb.3	9	+		171
chr6	33286904	33287054	AMPL87296	chr6	33286771	33286998	DAXX	uc011dre.2	1	-		94
chr6	33287262	33287479	AMPL87297	chr6	33287154	33287633	DAXX	uc011dre.2	2	-		217
chr6	33288610	33288783	AMPL87298	chr6	33288510	33289346	DAXX	uc011dre.2	5	-		173
chr6	33288925	33289140	AMPL87299	chr6	33288510	33289346	DAXX	uc011dre.2	5	-		215
chr7	41729195	41729399	AMPL87300	chr7	41728598	41730142	INHBA	uc003thr.3	0	-		204
chr7	41729876	41730087	AMPL87301	chr7	41728598	41730142	INHBA	uc003thr.3	0	-		211
chr7	42018104	42018305	AMPL87302	chr7	42018195	42018349	GLI3	uc011kbh.2	4	-		110
chr7	42087976	42088196	AMPL87303	chr7	42088087	42088297	GLI3	uc011kbh.2	10	-		109
chr7	55220129	55220348	AMPL87304	chr7	55220236	55220359	EGFR	uc003tqk.3	5	+		112
chr7	106512972	106513186	AMPL70423	chr7	106512979	106513049	PIK3CG	uc003vdw.3	2	+		70
chr7	106512972	106513186	AMPL70423	chr7	106513155	106513385	PIK3CG	uc003vdw.3	3	+		31
chr7	116380813	116381018	AMPL70352	chr7	116380903	116381081	MET	uc010lkh.3	4	+		115
chr7	140452977	140453136	AMPL40612	chr7	140453072	140453195	BRAF	uc003vwc.4	3	-		64
chr7	151877957	151878176	AMPL87308	chr7	151877793	151879681	KMT2C	uc003wla.3	23	-		219
chr7	151878887	151879030	AMPL87309	chr7	151877793	151879681	KMT2C	uc003wla.3	23	-		143
chr7	151879113	151879331	AMPL87310	chr7	151877793	151879681	KMT2C	uc003wla.3	23	-		218
chr7	151884181	151884400	AMPL87311	chr7	151884344	151884563	KMT2C	uc003wla.3	26	-		56
chr7	151962029	151962233	AMPL87312	chr7	151962120	151962296	KMT2C	uc003wla.3	51	-		113
chr8	38269877	38270027	AMPL70354	chr8	38268653	38271324	FGFR1	uc011lbu.2	0	-		150
chr8	38271537	38271759	AMPL70355	chr8	38271433	38271543	FGFR1	uc011lbu.2	1	-		6
chr8	38271537	38271759	AMPL70355	chr8	38271667	38271809	FGFR1	uc011lbu.2	2	-		92
chr8	38271883	38272102	AMPL87315	chr8	38272074	38272149	FGFR1	uc011lbu.2	3	-		28
chr8	38314716	38314934	AMPL70357	chr8	38314871	38315054	FGFR1	uc011lbu.2	16	-		63
chr9	21974485	21974696	AMPL87317	chr9	21974674	21975134	CDKN2A	uc003zpk.3	2	-		22
chr9	87342545	87342679	AMPL70360	chr9	87342566	87342876	NTRK2	uc004anz.1	10	+		113
chr9	101890939	101891146	AMPL87319	chr9	101891134	101891384	TGFBR1	uc004azc.3	1	+		12

chr9	101904688	101904877	AMPL87320	chr9	101904815	101904987	TGFBR1	uc004azc.3	4	+	62
chr9	101911351	101911534	AMPL87321	chr9	101911459	101916475	TGFBR1	uc004azc.3	8	+	75
chrX	44928833	44929045	AMPL87414	chrX	44928821	44929604	KDM6A	uc004dge.4	16	+	212
chrX	44948836	44949041	AMPL87415	chrX	44948985	44949177	KDM6A	uc004dge.4	24	+	56
chrX	44949782	44949971	AMPL70396	chrX	44949965	44950111	KDM6A	uc004dge.4	25	+	6
chrX	47039687	47039906	AMPL87417	chrX	47039608	47039710	RBM10	uc004dhi.3	10	+	23
chrX	47039687	47039906	AMPL87417	chrX	47039815	47039907	RBM10	uc004dhi.3	11	+	91
chrX	47041522	47041726	AMPL4592	chrX	47041558	47041727	RBM10	uc004dhi.3	16	+	168
chrX	47045096	47045247	AMPL87419	chrX	47045112	47045191	RBM10	uc004dhi.3	20	+	79
chrX	47045292	47045482	AMPL87420	chrX	47045461	47045572	RBM10	uc004dhi.3	21	+	21
chrX	47424368	47424587	AMPL70503	chrX	47424381	47424540	ARAF	uc004dic.2	4	+	159
chrX	66943411	66943558	AMPL66064	chrX	66943525	66950463	AR	uc004dwu.2	7	+	33
chrX	70356221	70356437	AMPL70397	chrX	70356128	70356507	MED12	uc004dyy.3	36	+	216
chrX	70360460	70360666	AMPL70455	chrX	70360482	70360709	MED12	uc004dyy.3	41	+	184
chrX	76777689	76777895	AMPL70504	chrX	76777738	76777868	ATRX	uc004ecp.4	3	-	130

Note:
The canonical exon genomic sequences (hg19) were downloaded from UCSC; the intersection between the exon transcripts and the designed amplicons was computed by bedtools.

Supplementary Table 3: allelic dropout calculation

sample_code	number_of_germline_SNPs	mean_allelic-dropout(ADO)_rate
PR01-1	13	0.211904418
PR01-2	13	0.165911935
PR01-3	11	0.199368483
PR02-1	15	0.1225017
PR02-2	15	0.192136752
PR02-3	15	0.147136812
PR02-4	15	0.187457283
PR03-1	14	0.234942138
PR03-2	14	0.247376657
PR03-3	15	0.155859138
PR03-4	15	0.187700464
PR04-1	12	0.20059493
PR04-2	12	0.183786321
PR04-3	12	0.240878658
PR05-1	11	0.165664317
PR05-2	12	0.203931292
PR05-3	13	0.191770432
PR05-4	11	0.194902719
PR05-5	13	0.281923896

Supplementary Table 4: subsampling experiment results

sample code	subsampled number of reads (million)	FASTQ size (R1, Gb)	Tapestri pipeline run time (hr)	max memory (Gb)	max processes	max threads	% reads mapped to panel	number of total barcodes (million)	% DNA read pairs assigned to cells	number of cells called	read depth (mean reads/cell/amplicon)	number of KRAS-mutated cells	% KRAS-mutated nuclei
PA04-2-frozen	50	2.6	3.92	21	62	964	90.36	0.88	27.740	1898	39	1732	91.25
PA04-2-frozen	100	5.1	6.64	25	62	962	90.36	1.21	32.630	2515	69	2305	91.65
PA04-2-frozen	150	7.5	10.55	27	62	964	90.36	1.40	34.380	2794	99	2570	91.98
PA04-2-frozen	200	9.8	20.48	27	62	963	90.36	1.53	35.400	2962	128	2729	92.13
PA04-2-frozen	250	12	17.81	27	62	963	90.36	1.63	36.900	3234	153	2979	92.12
PA04-2-frozen	278	17	N/A	N/A	N/A	N/A	90.36	1.67	37.120	3278	169	3012	91.89
PR05-3	50	2.7	5.47	21	62	964	92.20	0.95	60.590	2755	59	12	0.44
PR05-3	100	5.2	8.62	24	62	964	92.20	1.27	64.860	3199	109	20	0.63
PR05-3	150	7.7	12.02	27	62	964	92.20	1.46	66.070	3348	159	35	1.05
PR05-3	200	10	17.73	27	62	963	92.20	1.58	67.060	3491	206	49	1.40
PR05-3	250	13	18.23	28	62	964	92.20	1.67	67.580	3567	254	49	1.37
PR05-3	341	22	N/A	N/A	N/A	N/A	92.20	1.79	68.120	3651	342	82	2.25

Supplementary Methods

1 Doublet rate estimation

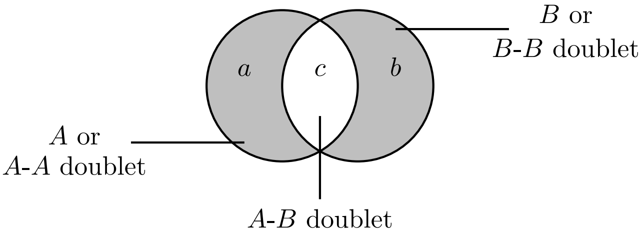

Supplementary Figure 11: Venn diagram showing the different possible observations and illustrating that it is not possible to distinguish between singlets and self-doublets.

Doublets occur when multiple cells get encapsulated in the same drop and acquire the same barcode. In microfluidic experiments the doublet rate δ, expected proportion of doublets in the data, depends on the rate at which the cells flow through the nozzle and time-interval in which a droplet is formed. When these quantities are small, the probability a doublet occurring can be approximated by a Binomial distribution [1–3] with a success probability δ.

We start with two cell lines A and B with unknown proportions x and y, respectively. Since there are only two cell lines in the mixture, we have $x + y = 1$. Let n be the total number of observations, out of which a contain only mutations from cell line A, b contain only mutations from cell line B and c contain mutations from both cell lines A and B (see **Supplementary Figure 11**). As such, $a + b + c = n$. Our goal is to compute the doublet rate δ for given values of a, b and c.

We assume that the number of observation is much smaller than the size of the initial mixture from which the cells are sampled. The expected total number of singlets is $n(1 - \delta)$, with number of singlets of cell line A is $x(1 - \delta)n$ and the number of singlets of cell line B is $y(1 - \delta)n$. Moreover, we assume that each cell in the doublet is picked independently from the mixture. A doublet can either be a neotypic A-B doublet, comprising of one cell from cell line A and one cell from cell line B, or a self-doublet A-A or B-B comprising of two cells from the cell line A or B, respectively. The expected number of A-A doublets is $x^2\delta n$, the expected number of B-B doublets is $y^2\delta n$ and the expected number of A-B doublets is $2xy\delta n$. Note that the expected total number of doublets is $n\delta$.

We need to solve the following set of nonlinear equations:

$$xn(1-\delta) + x^2\delta n = a \tag{1}$$

$$yn(1-\delta) + y^2\delta n = b \tag{2}$$

$$2xy\delta n = c, \tag{3}$$

$$a + b + c = n. \tag{4}$$

We solve these nonlinear equations numerically using the open-source package SCIPY.

2 Single-Cell Genotyper

We applied Single-Cell Genotyper (SCG) to case PA04's 3 multiregional samples, which include 1 primary tumor (PA04-1), 2 different sites of liver metastasis each with 2 technical replicates (PA04-2-fresh, PA04-2-frozen, PA04-3-fresh, PA04-3-frozen).

The clustering algorithm was chosen because it allows for:

- variable number of data states, which enables differentiating among heterozygous, homozygous, and missing SNV genotypes.
- variable layers of data, which enables inclusion of both SNV and CNV events.

without making any assumption of the evolutionary relationships among clusters.

2.1 input setup

For the SNV input matrix, we defined 4 states:

- 0 as wildtype (**WT**);
- 1 as heterozygous (**HET**);
- 2 as homozygous (**HOM**);
- 3 as missing (**MISS**);

In addition to the 4 somatic SNVs mentioned above, we also included germline SNPs validated by bulk on the matched normal sample of this patient (data included in **Supplementary Data**), since their SNV genotype could represent CNV events. The genotype of SNV in each single nucleus was assigned based on hard thresholds:

1. For an SNV to be considered **HET** in a cell, it needs to satisfy:
   (1) alternative allele read count > 0
   (2) variant allele frequency (VAF) > 0.2
2. For an SNV to be considered **HOM** in a cell, in addition to the two requirements for HET above, it needs to satisfy:
   (3) VAF > 0.8
3. For an SNV to be considered **MISS** in a cell, it needs to satisfy:
   (1) total read depth $= 0$

The thresholds were intentionally set leniently (except for **MISS**) because we hoped to push the error correction to SCG.

For the CNV input matrix, to avoid the complexity of accurate copy number calling which is not in the scope of this paper, we only included the homozygous deletion status:

- 0 as ploidy>0;
- 1 as ploidy=0.

This was based on our assumption that a read count of 0 very likely (99% as defined in the priors) represents a real homozygous deletion and likewise for a read count of nonzero.

Ploidy status was determined by a hard threshold, too: for an amplicon to be ploidy=0, it needs to satisfy that the forward read count strictly equals 0.

To focus on studying SMAD4's CNV evolution, we only included SMAD4's 8 amplicons. He had the hypothesis from observing the raw data (**Supplementary Figure 9**) that the 8 amplicons underwent a likely unsynchronous, step-wise process to the final state of 8/8 homozygous deletion, assuming that a deleted DNA cannot be regained.

We concatenated 8061 cells from all samples (including 2 technical replicates) of patient PA04 to do the clustering. The input matrices and the run parameters in YAML format are included in **Supplementary Data.**

2.2 running the clustering

2.2.1 model selection

We set the number of clusters to 40, with the belief that clonal selection would be almost complete in such a late-stage PDAC case and the number of observable/computationally solvable clone with respect to the input loci/regions should be less than 40.

2.2.2 hyperparameters

input state emission density (γ): Exactly as set up in the original SCG paper, we assume that data observed for each SNV/CNV is noisy, such that given the true genotype/ploidy state of loci is s, the probability of observing a value t is given by $\varepsilon_s \in$ [0,1], which can be modeled by a Dirichlet distribution with hyperparameters γ_s:

$$\varepsilon_s \mid \gamma_s \sim \text{Dirichlet}(\varepsilon_s \mid \gamma_s)$$

These hyperparameters were set as shown in **Supplementary Table 5** below, based on our understanding of the noise profile of the data.

cluster proportions: we set $\kappa = 0.01$, which controls the distribution of the cluster proportions.

doublet: We disabled modeling for doublets to improve the computational efficiency, given that the doublet rate had been estimated to be low.

$s \backslash t$	WT	HET	HOM	MISS
WT	88.9	1	0.1	10
HET	10	79	10	1
HOM	0.1	1	88.9	10
MISS	1	0.01	1	97.99

(a) SNV state parameters

$s \backslash t$	ploidy = 0	ploidy > 0
ploidy = 0	99	1
ploidy > 0	1	99

(b) CNV state parameters

Supplementary Table 5: Parameter settings for the *gamma* parameter for SNV and CNV data types. The rows correspond to hidden states and the columns correspond to observed states. Values are pseudo-counts in the Dirichlet distribution for s. Each row thus represents a setting of γ_s, the probability of observing t given s.

2.2.3 assessing convergence

After running SCG with 5000 random restarts, we recognize that the model is highly subject to local maxima. See below (**Supplementary Figure 12**) for the comparison of the evidence lower bound (ELBO) plots of 2000 and 5000 random restarts. The max ELBO improved very minimally and did not seem to be converging. Therefore, we did not move upwards from 5000 restarts.

2.3 building a phylogeny with inferred clusters

With the clustering returning only 7 distinct clusters for PA04, we thought inferring a clone tree was trivial: we defined a “genomic evolution cost” matrix for every SNV and CNV state transitions between each pair of unique clusters (**Supplementary Table 6**), and inferred the minimum spanning arborescence using a modified Edmond’s algorithm implemented in the NetworkX Python package [4].

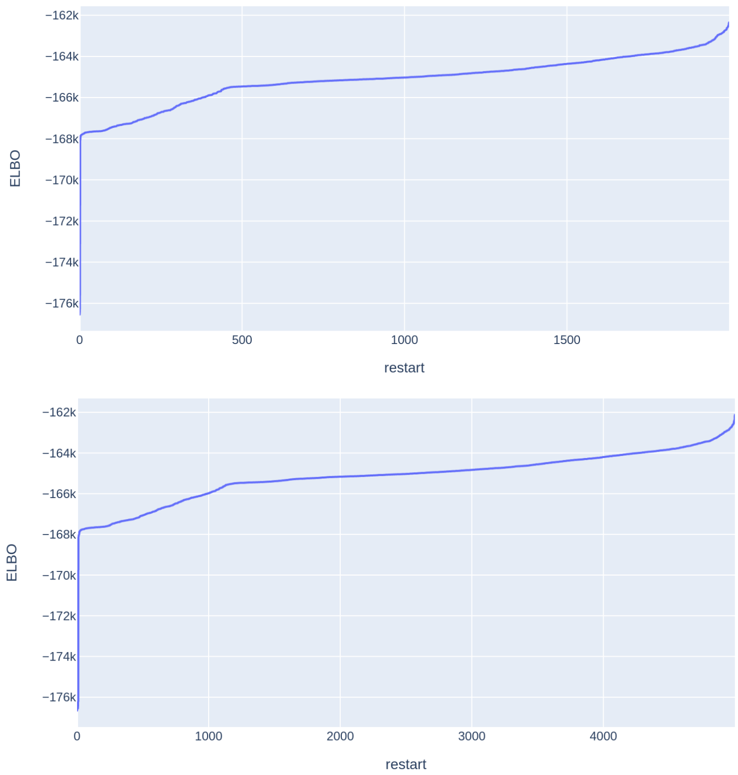

Supplementary Figure 12: ordered ELBO plots of 2000 and 5000 random restarts of SCG

	child				
parent		WT	HET	HOM	MISS
	WT	NaN	1	2	3
	HET	1	NaN	1	2
	HOM	2.5	∞	NaN	2
	MISS	∞	∞	∞	NaN

(a) SNV state transition costs

	child		
parent		ploidy $= 0$	ploidy > 0
	ploidy $= 0$	NaN	∞
	ploidy > 0	2	NaN

(b) CNV state transition costs

Supplementary Table 6: SNV and CNV state transition costs

Supplementary References

[1] Hamim Zafar, Anthony Tzen, Nicholas Navin, Ken Chen, and Luay Nakhleh. Sifit: inferring tumor trees from single-cell sequencing data under finite-sites models. *Genome biology*, 18(1): 1–20, 2017.

[2] Samuel L Wolock, Romain Lopez, and Allon M Klein. Scrublet: computational identification of cell doublets in single-cell transcriptomic data. *Cell systems*, 8(4):281–291, 2019.

[3] Leah L Weber, Palash Sashittal, and Mohammed El-Kebir. doubletd: detecting doublets in single-cell dna sequencing data. *Bioinformatics*, 37(Supplement_1):i214–i221, 2021.

[4] Aric A. Hagberg, Daniel A. Schult, and Pieter J. Swart. Exploring network structure, dynamics, and function using networkx. In Gaël Varoquaux, Travis Vaught, and Jarrod Millman, editors, *Proceedings of the 7th Python in Science Conference*, pages 11 – 15, Pasadena, CA USA, 2008.
