# Supplementary material for: Application of high-throughput single-nucleus DNA sequencing in pancreatic cancer
Source: Nat Commun. 2023 Feb 10;14:749. doi: 10.1038/s41467-023-36344-z (PMC9918733; doi:10.1038/s41467-023-36344-z)
Supplement: Supplementary file 4 — Supplementary Data 1-3 [file 41467_2023_36344_MOESM4_ESM.zip › Supplementary_Data/Supplementary_Data_Legend.docx]

**Supplementary Data Legend**

**Supplementary Data 1**

**a:** Case PA04’s tumor samples’ matched bulk whole-genome sequencing (WGS) results in mutation annotation format (MAF). The variant records were subset to our Tapestri panel’s genomic region. PA04-1: the identical piece of tissue was used for this primary pancreas tumor; PA04-2 and PA04-3: a different region of this liver metastasis was used.

**b:** Case PA04’s normal sample (PA04-0)’s bulk WGS result, processed from variant calling format (VCF) into allelic read counts. Variant records were subset to our Tapestri panel’s genomic region.

**Supplementary Data 2**

**a-j:** Case PR01-05’s tumor samples’ matched bulk whole-exome sequencing (WES) results in MAF format. The variant records were subset to our Tapestri panel’s genomic region. Note for each sample, the “SOMATIC” file corresponds to the “filtered” variant list while the “FILLOUT” file corresponds to the “unfiltered” variant list, as described in section “Bulk WES, WGS library preparation, sequencing, and variant calling” of **Methods.**

**Supplementary Data 3**

**a-c.** input matrices and single-cell-sample mapping files for single-cell genotyper (SCG).

**d.** run parameters in YAML format used for SCG.
