# Supplementary material for: Application of high-throughput single-nucleus DNA sequencing in pancreatic cancer
Source: Nat Commun. 2023 Feb 10;14:749. doi: 10.1038/s41467-023-36344-z (PMC9918733; doi:10.1038/s41467-023-36344-z)
Supplement: Supplementary file 5 — Reporting Summary [file 41467_2023_36344_MOESM5_ESM.pdf]

nature portfolio

Corresponding author(s): Christine Iacobuzio-Donahue
Haochen Zhang

Last updated by author(s): 10/21/2022

Reporting Summary

Nature Portfolio wishes to improve the reproducibility of the work that we publish. This form provides structure for consistency and transparency in reporting. For further information on Nature Portfolio policies, see our Editorial Policies and the Editorial Policy Checklist.

Statistics

For all statistical analyses, confirm that the following items are present in the figure legend, table legend, main text, or Methods section.

n/a	Confirmed	
☐	☒	The exact sample size (*n*) for each experimental group/condition, given as a discrete number and unit of measurement
☐	☒	A statement on whether measurements were taken from distinct samples or whether the same sample was measured repeatedly
☒	☐	The statistical test(s) used AND whether they are one- or two-sided<br>*Only common tests should be described solely by name; describe more complex techniques in the Methods section.*
☒	☐	A description of all covariates tested
☒	☐	A description of any assumptions or corrections, such as tests of normality and adjustment for multiple comparisons
☐	☒	A full description of the statistical parameters including central tendency (e.g. means) or other basic estimates (e.g. regression coefficient) AND variation (e.g. standard deviation) or associated estimates of uncertainty (e.g. confidence intervals)
☒	☐	For null hypothesis testing, the test statistic (e.g. *F*, *t*, *r*) with confidence intervals, effect sizes, degrees of freedom and *P* value noted<br>*Give P values as exact values whenever suitable.*
☒	☐	For Bayesian analysis, information on the choice of priors and Markov chain Monte Carlo settings
☒	☐	For hierarchical and complex designs, identification of the appropriate level for tests and full reporting of outcomes
☒	☐	Estimates of effect sizes (e.g. Cohen's *d*, Pearson's *r*), indicating how they were calculated

Our web collection on statistics for biologists contains articles on many of the points above.

Software and code

Policy information about availability of computer code

Data collection	No software was used to collect the data.
Data analysis	Bulk data variant calling:<br>- MSKCC bioinformatics core variant calling pipeline (available at https://github.com/soccin/BIC-variants_pipeline), which includes: bwa mem v0.7.12, GATK v3.1 (mutect v1.1.6, HaplotypeCaller v2.4).<br><br>Mission Bio Tapestri pipeline (v2.0.1): currently not publically availble.<br>DAb-seq pipeline (similar enough to the Mission Bio Tapestri pipeline): https://github.com/AbateLab/DAb-seq.<br>Mission Bio Mosaic (the default package can be found at https://anaconda.org/missionbio): the customized version used is available at https://github.com/haochenz96/mosaic (frozen branch: "ncomm_2022_publication").<br>Tapestri Express Analysis (TEA), available at https://github.com/haochenz96/tea (frozen branch: "ncomm_2022_publication").<br>Single-cell Genotyper (SCG): https://github.com/haochenz96/scg

For manuscripts utilizing custom algorithms or software that are central to the research but not yet described in published literature, software must be made available to editors and reviewers. We strongly encourage code deposition in a community repository (e.g. GitHub). See the Nature Portfolio guidelines for submitting code & software for further information.

Data

Policy information about availability of data

All manuscripts must include a data availability statement. This statement should provide the following information, where applicable:

- Accession codes, unique identifiers, or web links for publicly available datasets
- A description of any restrictions on data availability
- For clinical datasets or third party data, please ensure that the statement adheres to our policy

The hg19 reference genome used in this study came from UCSC (http://hgdownload.cse.ucsc.edu/goldenpath/hg19/bigZips/) and Broad Institute (https://gatk.broadinstitute.org/hc/en-us/articles/360035890711-GRCh37-hg19-b37-humanG1Kv37-Human-Reference-Discrepancies#b37).

Sequencing data have been deposited at the European Genomephenome Archive (EGA), which is hosted by the European Bioinformatics Institute and the Centre for Genomic Regulation, under accession number EGAS00001006024. Further information about EGA can be found at https://ega-archive.org and "The European Genomephenome Archive of human data consented for biomedical research" (http://www.nature.com/ng/journal/v47/n7/full/ng.3312.html). These data include:
- Raw FASTQ files for each Tapestri sample.
- BAM files output by the default Mission Bio Tapestri pipeline for each sample.
- HDF5 files output by the default Mission Bio Tapestri pipeline for each sample.

The bulk WES data used in Figure 2 and Supplementary Figure 4 are provided in the Supplementary Information file. Note these are already subset to our panel's genomic region. Any other data can be made available upon request. Source data are provided with this paper.

Field-specific reporting

Please select the one below that is the best fit for your research. If you are not sure, read the appropriate sections before making your selection.

☒ Life sciences ☐ Behavioural & social sciences ☐ Ecological, evolutionary & environmental sciences

For a reference copy of the document with all sections, see nature.com/documents/nr-reporting-summary-flat.pdf

Life sciences study design

All studies must disclose on these points even when the disclosure is negative.

Sample size	No sample size calculation was performed. In this study, 38 snDNA libraries were generated, which include 34 biologically distinct snap frozen pancreatic adenocarcinoma (PDAC) samples from 16 individual patients. 4 of the samples had one technical replicate each. Each individual snDNA library originated from one individual nuclei extraction run and Tapestri microdroplet-based library preparation. The sample size was deemed sufficient because the sample cohort reflected a good spectrum of technical variations one may encounter while sequencing PDAC samples- different tissue origins (primary site vs metastatic site), different stages of disease (resectable vs metastatic), different total cellularity and tumor purity.
Data exclusions	No data was excluded from analysis.
Replication	The 38 distinct snDNA libraries work as replicates to validate the effectiveness of our single nuclei preparation method.<br>For the cryopreservation experiment, we had two distinct nuclei samples each cryopreserved for different lengths of time and analyzed against their fresh counterpart in terms of the resulting snDNA library.<br><br>All replication experiments described in the manuscript were successful.
Randomization	The 34 biologically distinct samples were chosen randomly except to reflect the technical variations with respect to single nuclei DNA sequencing library preparation, as described above. We do not believe any covariates (e.g. patient's biological profile) were relevant for that purpose.<br>The 2 samples used for nuclei cryopreservation experiment were chosen randomly from samples with sufficient nuclei extracted. Again, since this is to test the sample processing workflow, we do not believe any covariates, such as the biological properties of the samples, are relevant.
Blinding	Because we are using patient tissue sample rather than conducting experiments on patients directly, blinding was not applicable.

Reporting for specific materials, systems and methods

We require information from authors about some types of materials, experimental systems and methods used in many studies. Here, indicate whether each material, system or method listed is relevant to your study. If you are not sure if a list item applies to your research, read the appropriate section before selecting a response.

Materials & experimental systems

n/a	Involved in the study
☒	☐ Antibodies
☒	☐ Eukaryotic cell lines
☒	☐ Palaeontology and archaeology
☒	☐ Animals and other organisms
☐	☒ Human research participants
☒	☐ Clinical data
☒	☐ Dual use research of concern

Methods

n/a	Involved in the study
☒	☐ ChIP-seq
☒	☐ Flow cytometry
☒	☐ MRI-based neuroimaging

Human research participants

Policy information about studies involving human research participants

Population characteristics	The genotypic information of our patient cohort is described in Figure 1c. We do not believe any other covariates are relevant because we did not attempt to draw any population-level biological conclusions, but rather focused on individual cases.
Recruitment	Autopsy: patients agreed to donate their tissues to ongoing cancer research after they passed away through the medical donation program at Memorial Sloan Kettering Cancer Center or the Rapid Autopsy Program program at Johns Hopkins Medicine.<br>Resection/biopsy-derived organoid: patients provided informed consent prior to tissue acquisition at Memorial Sloan Kettering Cancer Center.<br>All patients provided written consent and were not compensated.<br>We do not find any potential bias caused by patient recruitment, because this study's purpose is largely proof-of-principle for the single nucleus DNA sequencing library preparation workflow, and the biological insights drawn were all based on individual case studies.
Ethics oversight	Institutional Review Board at Memorial Sloan Kettering Cancer Center and Johns Hopkins Medicine.

Note that full information on the approval of the study protocol must also be provided in the manuscript.
